# Structural and functional insights into the first *Bacillus thuringiensis* vegetative insecticidal protein of the Vpb4 fold, active against western corn rootworm

**DOI:** 10.1371/journal.pone.0260532

**Published:** 2021-12-20

**Authors:** Jean-Louis Kouadio, Meiying Zheng, Michael Aikins, David Duda, Stephen Duff, Danqi Chen, Jun Zhang, Jason Milligan, Christina Taylor, Patricia Mamanella, Timothy Rydel, Colton Kessenich, Timothy Panosian, Yong Yin, William Moar, Kara Giddings, Yoonseong Park, Agoston Jerga, Jeffrey Haas

**Affiliations:** 1 Bayer Crop Science, Chesterfield, Missouri, United States of America; 2 Department of Entomology, Kansas State University, Manhattan, Kansas, United States of America; University of Texas at Dallas, UNITED STATES

## Abstract

The western corn rootworm (WCR), *Diabrotica virgifera virgifera* LeConte, is a major maize pest in the United States causing significant economic loss. The emergence of field-evolved resistant WCR to *Bacillus thuringiensis* (*Bt*) traits has prompted the need to discover and deploy new insecticidal proteins in transgenic maize. In the current study we determined the crystal structure and mode of action (MOA) of the Vpb4Da2 protein (formerly known as Vip4Da2) from *Bt*, the first identified insecticidal Vpb4 protein with commercial level control against WCR. The Vpb4Da2 structure exhibits a six-domain architecture mainly comprised of antiparallel β-sheets organized into β-sandwich layers. The amino-terminal domains 1–3 of the protein share structural homology with the protective antigen (PA) PA14 domain and encompass a long β-pore forming loop as in the clostridial binary-toxB module. Domains 5 and 6 at the carboxyl-terminal half of Vpb4Da2 are unique as this extension is not observed in PA or any other structurally-related protein other than Vpb4 homologs. These unique Vpb4 domains adopt the topologies of carbohydrate-binding modules known to participate in receptor-recognition. Functional assessment of Vpb4Da2 suggests that domains 4–6 comprise the WCR receptor binding region and are key in conferring the observed insecticidal activity against WCR. The current structural analysis was complemented by *in vitro* and *in vivo* characterizations, including immuno-histochemistry, demonstrating that Vpb4Da2 follows a MOA that is consistent with well-characterized 3-domain *Bt* insecticidal proteins despite significant structural differences.

## Introduction

The gram-positive soil bacterium, *Bacillus thuringiensis* (*Bt*), is a well-known source of insecticidal proteins, effectively utilized against insect pests from various orders. In the mid-1990s, the introduction of transgenic-crops utilizing *Bt* insecticidal proteins, and their rapid adoption by farmers across the United States (US) and elsewhere [1], has led to significant expansion of this technology. In 2020, 82 percent of the total maize, *Zea mays L*, acres in the US were planted with *Bt* transgenic maize https://www.ers.usda.gov/data-products/adoption-of-genetically-engineered-crops-in-the-us/recent-trends-in-ge-adoption.aspx.

The western corn rootworm (WCR), *Diabrotica virgifera virgifera* LeConte, and a related species, northern corn rootworm (NCR), *D*. *barberi*, Smith & Lawrence, are major pests of maize in North America [2, 3], causing significant damage by larvae feeding on maize roots [4]. To date, transgenic maize developed to control WCR and related *Diabrotica* species express *Bt*-derived three-domain crystal (3-D Cry) proteins of the δ-endotoxin structural class, namely Cry3Bb1 [5], mCry3A [6], and eCry3.1Ab [7]. These proteins utilize an α-helical bundle to form pores in the midgut cell membrane and are classified as bacterial alpha-pore forming proteins (α-PFPs). Additional *Bt* proteins targeting corn rootworm (CRW) larvae are the binary proteins Gpp34Ab1/Tpp35Ab1 (formerly known as Cry34Ab1/Cry35Ab1) [8]. These β-strand-rich proteins are structurally distinct from the classical 3-D Cry proteins, utilizing a β-hairpin secondary structure to form pores [9] and therefore are classified as a bacterial β-pore-forming protein (β-PFP).

Despite the progress in transgenic *Bt* crop-protection to CRW, field-evolved resistant WCR populations have been reported for Cry3Bb1 [10] and Gpp34Ab1/Tpp35Ab1 [11] maize. Additionally, Cry3Bb1 resistant WCR colonies have shown cross-resistance to mCry3A [12] and eCry3.1Ab [13] maize, threatening the longevity of all commercially available transgenic maize to control WCR. Therefore, it is critical to find new insecticidal proteins for below-ground insect protection of maize. The search for new insecticidal proteins to control WCR has recently resulted in the identification of the *Bt* insecticidal protein, Vpb4Da2, specifically active against WCR larvae [14]. This new Vpb4Da2 protein controls both Cry3Bb1 and Gpp34Ab1/Tpp35Ab1-resistant WCR colonies confirming that Vpb4Da2 MOA is unique likely due to differences in its WCR receptor utilization [14]. Amino acid sequence analysis designates Vpb4Da2 as a new β-PFP member of the Bacterial_exotoxin_B protein family (IPR035088) with Vpb4Da2 domain architecture composed of PA14 (PF07691) and Binary-toxB (PF03495) protein family (Pfam) domains. Proteins from the Bacterial_exotoxin_B family are non-toxic binding components (B-component) of the AB-toxin super-family [15] and include protective antigen (PA) [16], C2 component (C2-II) [17], Iota toxin component (Ib-component) [18], vegetative insecticidal protein 1, (Vpb1; formerly known as Vip1) [19] and other Vpb4’s [20]. The amino-terminal domains 1–3 of these proteins exhibit a conserved structural organization shared across the family whereas the carboxyl-terminal is comprised of at least one domain [21]. For PA, domain 1 contains a cysteine protease cleavage site integral to oligomerization and pre-pore formation, domain 2 contains a long stem region that changes conformation at acidic pH to form a β-barrel pore, and domain 3 is involved in oligomer formation [22]. The receptor binding region at the carboxyl-terminal end of the Binary-toxB protein encompasses one or more carbohydrate binding modules responsible for receptor-recognition [16, 18]. Vpb4Aa1 (formerly known as Vip4Aa1), identified by Sun *et al*. in 2010 (Accession number HM044666), was described as lacking insecticidal properties, hampering subsequent functional characterization.

The current study is the first to describe the structure-function relationship of a protein of the Vpb4 fold, Vpb4Da2, with insecticidal activity against WCR. Here we report on the crystal structure of Vpb4Da2, which displays structural homology to β-PFPs from the Bacterial_exotoxin_B family. Furthermore, the primary steps leading to its activity against WCR were found to be the same canonical MOA as the traditional *Bt* 3-domain Cry proteins, despite the lack of shared structural homology.

## Materials and methods

### Cloning, expression, and purification

Wild-type Vpb4Da2 (pMON346342), Vpb4C.6693 (pMON342343; a natural variant with 77% amino acid sequence identity to Vpb4Da2), truncated Vpb4Da2 variants, domain-swaps between Vpb4Da2 and Vpb4C.6693, and Vpb4Da2 cysteine variants were cloned into a T7 expression system and either synthesized using unimolecular and multipart hot fusion [23] or by a third party vendor (Bio Basic, INC). Mutations were confirmed using Sanger and next generation sequencing (Illumina).

Vpb4Da2, Vpb4C.6693, and their derivatives were expressed in *E*. *coli* Rosetta™ 2(DE3) cells using an auto-induction system as described by Studier [24]. Briefly, overnight cultures in Luria-Bertani (LB) liquid medium were used to seed an auto-induction medium supplemented with 100 μg/mL Kanamycin and 25 μg/mL Chloramphenicol. Cell growth and induction were performed at 37°C for 3 h and subsequently at 20°C for 44 h. Cells were harvested by centrifugation and resulting pellets frozen at -80°C until used. For protein purification, cell pellets were lysed for 30 min at 4°C with a 3:1 (vol/vol) mixture of B-PER™(Bacterial protein extraction reagent, Thermo Scientific) and Y-PER™ (Yeast protein extraction reagent, Thermo Scientific) supplemented with 25 mM Tris-HCl, pH 8.0, 200 mM NaCl, 0.1 mg/mL lysozyme, 250 units/mL benzonase (ART.sm nuclease expressed pMON101670), 2.5 mM MgCl_2_, 1 mM phenylmethylsulfonyl fluoride (PMSF), and 10 mM imidazole, clarified by centrifugation at 20,000 x g at 4°C for 15 min and the supernatant subjected to His-select™ nickel resin (MilliporeSigma) affinity purification. Nickel resin eluates were loaded onto a Superdex-200 column (Cytiva), equilibrated with 25 mM sodium carbonate, pH 9.5 and 25 mM NaCl, for size-exclusion chromatography at a flow rate of 1 mL/min using an AKTA Pure^TM^ fast protein chromatography (FPLC) system (Cytiva). Protein fractions were pooled and concentrated using Amicon® Ultra-15 centrifugal filters (MilliporeSigma). Protein concentrations were determined using absorbance at 280 nm and the molecular weight evaluated by intact molecular weight determination using a Q-Tof LC/MS spectrophotometer (Waters) [25]. Optimum protein yield for Vpb4C.6693 and chimera-2 was achieved using a one-step His-select™ nickel resin affinity purification, followed by two buffer exchanges using Zeba™ spin desalting columns, 7K MWCO (Thermo Scientific), equilibrated with 25 mM sodium carbonate pH 9.5 and 25 mM NaCl. Protein concentrations for these two proteins were determined by spot-densitometry using BSA as standard. In-gel digestion and mass spectrometric analyses adapted from Shevchenko et *al* [26] were used to confirm protein identity.

### Crystal structure determination

A surface entropy reduction approach was used to generate the variant Q530Y-E531Y-K532Y which was used for successful crystallization of Vpb4Da2. Purification of the Vpb4Da2 variant was performed as described above except that size-exclusion chromatography was performed using a Superdex-200 equilibrated with 25 mM Tris-HCl, pH 8.0, 150 mM NaCl, 0.5 mM EDTA, and 1 mM DTT. Purified protein at 10 mg/mL was subjected to crystallization trials using Wizard 1, 2, 3, and 4 (Rigaku reagents), PEGRx and Crystal Screen (Hampton Research), and the sitting drop vapor diffusion technique in 96-well trays. Vpb4Da2 crystals were obtained in 0.1 M Bis-Tris at pH 6.5, 1.8 M ammonium sulfate, and 2% (w/v) PEG MME 550. Crystals were optimized by varying the pH and ammonium sulfate concentration. Crystals were cryo-cooled using glycerol.

Data were remotely collected at the Southeast Regional Collaborative Access Team (SER-CAT) 22-ID beamline of the Advanced Photon Source (APS) at Argonne National Laboratory, and were processed using the HKL2000 package [27]. The structure was solved by molecular replacement phasing using an internal Vpb4-based phasing model and the Phenix package [28]. Iterative Phenix refinement and program Coot [29] map-fitting resulted in a reasonably good quality final model of the full-length structure, as assessed by MolProbity [30] scores. The final structure was deposited with the PDB/RCSB under accession code 7MJR.

### Insect bioassay and feeding

Eggs from susceptible non-diapausing WCR (Waterman) were used for all experiments. Artificial diet bioassays were performed using a 96-well plate containing southern corn rootworm (SCR) larval diet (Frontier Scientific F9757). Molten diet was dispensed at 200 μL per well. Protein samples (20 μL) were overlaid onto solidified diet surface and allowed to dry. One neonate larva (< 24 h post-hatch) was infested per well and plates were sealed using pre-punched heat sensitive seals. Plates were subsequently incubated in a dark environmental chamber at 27°C, 60% humidity. Except for the labeled protein feeding experiment as described below, all bioassays evaluated larval mortality relative to control samples six days post infestation. Larvae were considered dead if they were immobile when touched with a fine tip paintbrush. Any bioassay was discarded if more than 30% mortality or less than 70% infestation was detected on the water control, or more than 15% contamination was observed. Mortality was evaluated in a column-wise manner. Mortality for a column was calculated from the number of wells with dead larvae in the column.

### Competition bioassay analysis

Mass action *in vivo* competition bioassay was performed as previously described [31, 32]. Wild-type Vpb4Da2 (23.53 μg/cm^2^) was mixed with the disabled insecticidal protein (DIP) variant, Vpb4Da2-T295C_T493C, as a competitor at 23.53 μg/cm^2^ and at 235.30 μg/cm^2^. DIP variant alone was also tested at 235.30 μg/cm^2^. Assay buffer was 25 mM Na carbonate pH 10.5 and 25 mM NaCl. Protein mixtures were fed to neonate WCR using the larval diet bioassay described above.

### WCR gut fluid collection

Insect gut fluid collection was as described by Girard *et al* [33]. Briefly, dissected insect guts from about 10 third instar larvae, reared on maize isoline seedling roots, were mixed with 50 μL of ice-cold 150 mM NaCl and gently stirred on ice with a Teflon pestle. Midgut tissue and fluid were then centrifuged at 10,000 x *g* for 5 min at 4°C. Resulting supernatant (gut fluid) was carefully collected to avoid contamination from the fat layer. Protein concentration was determined using the Bradford assay (Bio-Rad), and the sample was stored at -80°C. Total Cathepsin B protease activity was assessed using the fluorogenic substrate Z-Arg-Arg-7-amino-methylcoumarin-HCl (MilliporeSigma).

### Protein labeling, fate assessment in WCR midgut, and *in vitro* processing

Double cysteine variants Vpb4Da2-T283C_T427C (used in this section below), Vpb4Da2-K733C_A422C, and Vpb4Da2-T295C_T493C (these last two double cysteine variants used in the “In vitro oligomer formation by Vpb4Da2” section below) were labeled in protein storage buffer (25 mM Na carbonate, pH 9.5 and 25 mM NaCl) with 0.5 mM 5-Iodoacetimido fluorescein (5-IAF) at room temperature for 30 min. Excess label was removed by buffer exchange in protein storage buffer using a Zeba™ spin desalting column, 7K MWCO (Thermo Scientific). Labeling efficiency was assessed by Q-TOF LC/MS (Waters), and protein concentration determined using the Bradford assay (Bio-Rad) using BSA as standard.

WCR neonates were fed with 23.53 μg/cm^2^ of fluorescently labeled Vpb4Da2-T283C_T427C on artificial diet for 24 h to evaluate the labeled protein fate in WCR midguts. Samples of 25 insects were collected on dry ice and frozen at -80°C until used. Samples were homogenized in 5 mM Tris-HCl at pH 7.4 supplemented with 1 mM PMSF (MilliporeSigma), 1 mg/mL cytidine 5’-dicytidine 5′-diphosphocholine sodium salt dihydrate (MilliporeSigma) as a lipase inhibitor, and a protease inhibitor cocktail (MilliporeSigma), using a 2 mL Dounce homogenizer. Homogenates were centrifuged at 21,000 x *g* for 10 min and resulting pellets solubilized in 100 μL PBS, pH 7.4, and 1 mM PMSF. Total protein concentration was determined using the Bradford assay and BSA as standard. Approximately 10 μg of the re-solubilized pellet sample was loaded onto SDS-PAGE and fluorescence imaging was obtained using a ChemiDoc MP imaging system (Bio-Rad).

*In vitro* protein processing with WCR gut fluid was performed at room temperature at a ratio of 4:1 protein:gut fluid (w/w) in 30 mM MES pH 6.0, 50 mM NaCl for up to 90 min and stopped with protease inhibitor cocktail (MilliporeSigma). Proteolytic processing of Vpb4Da2 was performed with L-(tosylamido-2-phenyl) ethyl chloromethyl ketone (TPCK) treated trypsin, (MilliporeSigma) at pH 9.5, at a 200:1 ratio protein:trypsin (w/w) and incubated at room temperature for 15 min or longer if needed. The reaction was stopped using 1 mM of PMSF for 30 min at room temperature. To determine the proteolytic cleavage sites within Vpb4Da2, intact molecular weight of proteolytic fragments were analyzed using Q-TOF LC/MS spectrophotometer (Waters) [25]. For insect bioassays with trypsin-processed Vpb4Da2, processed samples were further buffer-exchanged into bioassay buffer (25 mM NaCl, pH 10.0 and 25 mM NaCl) using a Zeba™ desalting column, 7 kDa MWCO (Thermo Scientific), and quantified by absorbance at 280 nm.

### Brush border membrane preparation

A modified brush border membrane (BBM) method described by English *et al*. [34] was adapted for isolating BBM from whole WCR neonates. Briefly, frozen (-80°C) WCR non-fed neonates, less than 24h old, were homogenized in 5 mM Tris-HCl pH 7.4, 50 mM sucrose supplemented with Cytidine 5’-diphosphocholine (MilliporeSigma), protease inhibitor cocktail (MilliporeSigma), and PMSF at 4°C using three 30 s bursts of a PT 2500E polytron homogenizer (Kinematica, Inc) at 15,000 rpm. One volume of 5 mM Tris-HCl pH 7.4, 50 mM sucrose was added to the homogenate, supplemented with 10 mM CaCl_2_, stirred on ice for 30 min and centrifugated at 4,500 x *g* for 30 min. The cleared lysate was passed through four layers of cheese cloth and further centrifuged at 27,000 x *g* for 30 min. The resulting pellet was re-suspended in half of the previous volume, homogenized on ice with a Dounce homogenizer (MilliporeSigma), supplemented with 10 mM CaCl_2_, and further centrifuged at 27,000 x *g* for 30 min. The BBM pellet was re-suspended in 0.32 M sucrose, aliquoted, flashed frozen in liquid nitrogen, and stored at -80°C. Total protein concentration was determined by using the Bradford assay (Bio-Rad) with bovine serum albumin (BSA) as a standard. Enrichment was determined using the enzyme biomarker aminopeptidase-N (APN) and leucine-p-nitroanilide (MilliporeSigma) as substrate [35]. BBM samples showing 23 to 36-fold enrichment were used subsequently in binding assays.

### *In vitro* oligomer formation by Vpb4Da2

The fluorescently-labeled variant, Vpb4Da2-K733C_A422C, was subjected to trypsin processing, and its oligomeric state assessed in various pH solutions (25 mM Na citrate pH 5.0 and 25 mM NaCl, 25 mM Na phosphate pH 6.0 and 25 mM NaCl, and 25 mM Na carbonate pH 9.0 and 25 mM NaCl). The Vpb4Da2 oligomer reaction was conducted at room temperature with 0.05 mg/mL protein for 1 h in the presence of 10 mM of the homo-bifunctional cross-linker, bis (sulfosuccinimidyl)suberate (BS3) (Thermo Scientific). The crosslink reaction was quenched with 20 mM Tris base. Samples were evaluated using a 4–20% SDS-PAGE (Bio-Rad) and imaged on a Chemidoc^™^ MP imager (Bio-Rad) using an excitation/emission of 470/605 nm.

The SDS-resistant Vpb4Da2 oligomer evaluation was performed with trypsin-processed and fluorescently-labeled Vpb4Da2-K733C_A422C (insecticidal) and Vpb4Da2-T295C_T493C (pore-forming disabled and non-insecticidal). The reaction was conducted with 0.05 mg/mL protein and 5 μg WCR BBMV, at room temperature or at 37°C, for 1 h, at various pH values. Samples were separated using SDS-PAGE and imaged as described above.

### Immuno-histochemistry

Neonate WCR larvae were collected at designated time points after feeding on diet that was surface-treated with 23.53 μg/cm^2^ Vpb4Da2. Larvae were dissected for gut morphology assessment. Paraffin sections were obtained by fixing entire larvae in phosphate buffer saline (PBS), pH 6.9, containing 4% (w/v) paraformaldehyde and incubated at 4°C overnight. Samples were first washed in PBS containing 0.5% (v/v) Triton X-100 (PBST) and subsequently dehydrated in a series of 20 min incubation steps with 50%, 75%, and 95% (v/v) ethanol (in water). Dehydrated samples were further incubated in chloroform at room temperature for 4 h and in liquified paraffin at ~ 60°C overnight. Paraffin blocks were cut to 5 μm thick slices, deparaffinized in xylene for 10 min, and rehydrated by incubating slices in a series of 5 min wash steps using 100%, 96%, 70%, 40% and 0% (v/v) ethanol (in ddH_2_O), followed by a PBST wash for 10 min.

For immuno-blotting and staining, samples were blocked with 10% (v/v) normal goat serum (NGS) in PBST for 40 min and rinsed 3 times with PBST. Primary anti-rabbit polyclonal antibody against Vpb4Da2 was applied at 1:100 dilution in PBST at 4°C overnight. After 3 washes with PBST (5 min each), sections were incubated with a goat anti-rabbit IgG secondary antibody conjugated with Alexa Fluor 488 (Jackson Immuno-Research Laboratories, INC.) in the dark at room temperature for 4 h. Sections were subsequently washed three times (5 min each) in PBST and mounted in FluoroQuest Mounting Medium (AAT Bioquest) containing 4’,6’Diamidine-2’-phenylindole dihydrochloride (DAPI). Images were captured using a confocal microscope (Zeiss LSM 700).

### Bioinformatic analysis

The degree of conservation for each Vpb4Da2 amino acid was obtained using the *Consurf* server [36]. The Vpb4Da2 (AZJ95709.1) amino acid sequence was used as template to query the *Consurf* server [37]. An iterative search against the UNIREF-90 database selected 89 Vpb4Da2 sequence homologs and provided the amino acid conservation score at each position, which was used to map sequence conservation onto the Vpb4Da2 surface. This was accomplished with a custom R code and the bio3D R package [38] to map and write the sequence conservation scores into the Vpb4Da2 PDB file in place of B-factors. The structure was colored based on the conservation score using PyMOL™2.02.

Tree building was conducted using MEGA X [39] following methods outlined for the broader *Bt* Nomenclature [40] and consisted of a tree inferred using the UPGMA method with 1000 bootstrap replicates computed using the Dayhoff substitution model. This analysis involved 12 sequences and was conducted using the pairwise deletion option resulting in 1035 positions in the dataset. Branch tips were annotated using protein identifiers and the global percent identity based on a pairwise comparison to protective antigen (AAA22637.1). Pairwise alignments were conducted with the ggsearch36 algorithm available as part of the FASTA 36.3.8g package [41]. Color blocks were generated using a custom script to convert raw alignment output into blue (identical/conserved), grey (similar/semi-conserved), and red (mismatch/gap) blocks based on underlying alignment data.

### Analytical assessments

Analytical size-exclusion chromatography (SEC) of the full-length and trypsin-processed Vpb4Da2 was performed on an AKTA pure™ using a Superdex-200 10/300 GL increase (Cytiva) equilibrated at 4°C with 25 mM Na carbonate pH 9.5 and 100 mM NaCl. Protein samples were injected at the flow rate of 0.5 mL/min. Superdex-200 10/300 was calibrated using Ferritin (440, 000 Da), Aldolase (158, 000 Da), Conalbumin (75,000 Da), Ovalbumin (44, 000 Da), and Blue dextran 2000, which were all from Cytiva. K*av* = (*V*e-*V*o)/*V*c-*V*o), with *V*e = elution volume, *V*o = void volume (8.07 mL), and *V*c = Column volume (24 mL) were used. Calibration curve equation, Y = -03896x + 1.2061 (R^2^ = 0.9225) was used to calculate the molecular weight for full length and trypsin processed Vpb4Da2.

Thermal stability of the purified wild-type Vpb4Da2 and variants in protein storage buffer was performed in triplicate reactions using 25 μL of 0.5 mg/mL protein and 5 μL of 10x SYPRO™ Orange (Thermo Scientific). The CFX96™ real-time PCR detection system (Bio-Rad) was used to follow a thermal denaturation gradient of 20°C to 95°C with an increment of 0.5°C per 5 Sec. Fluorescence data were fit using the CFX manager 2.1 (Bio-Rad).

## Results

### Vpb4Da2 structure

**Fig 1:**
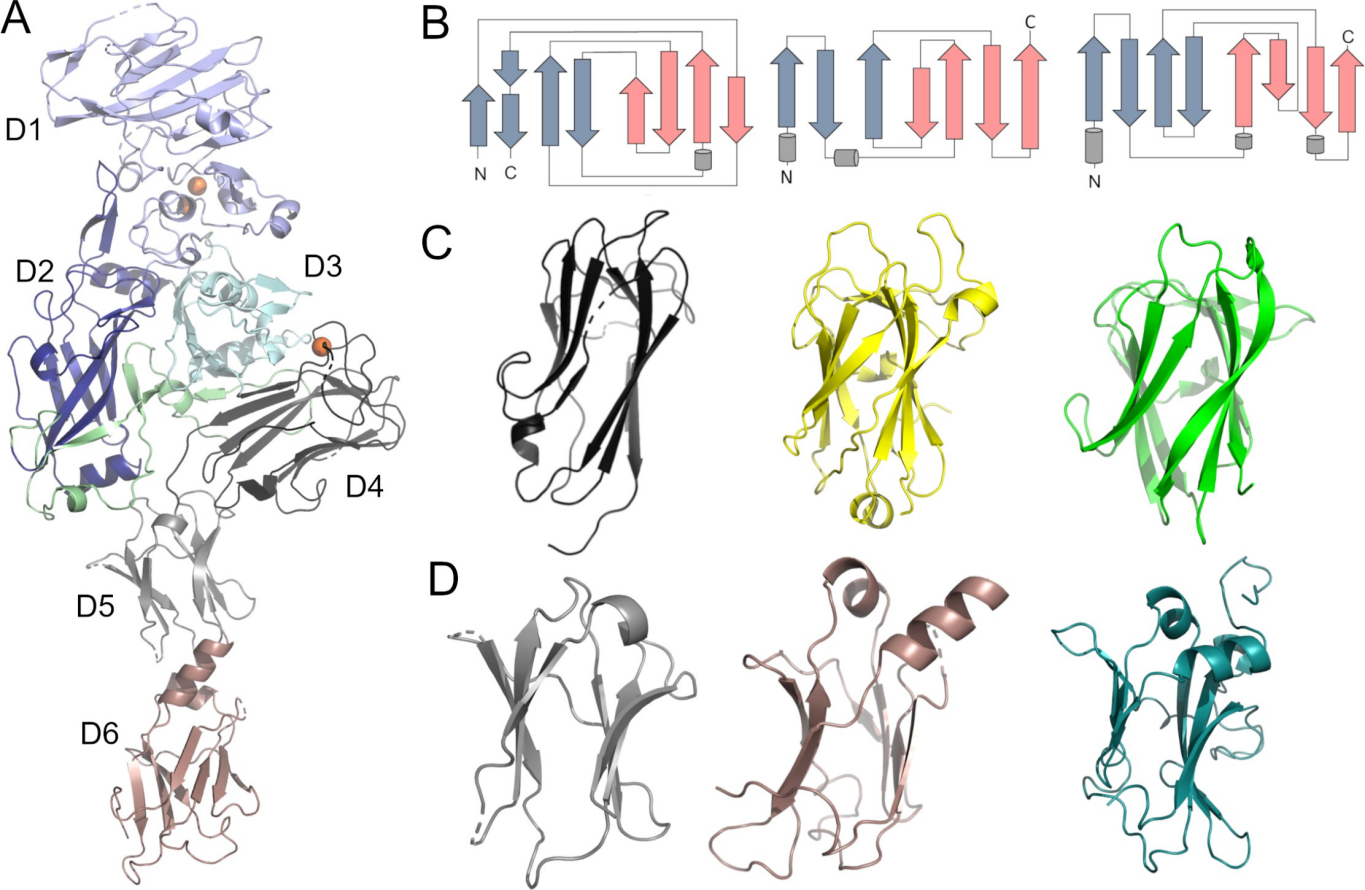
*Bacillus thuringiensis* Vpb4Da2 crystal structure. **(A)** Crystal structure of Vpb4Da2 structure and domains labeled D1 to D6. Predicted pore-forming loop is in pale green and calcium ions are orange spheres. **(B)** Topology diagrams of Vpb4Da2 domain 4 (left) and domain 6 (center) versus PA domain 4 from PDB ID Acc1 (right). **(C)** Vpb4Da2 domain 4 (left) versus Cry3Bb1 domain 3 from PDB ID 1ji6 (center) and *Clostridium thermocellum* xylanase carbohydrate binding module from PDB ID 1gmm (right). **(D)** Vpb4Da2 domains 5 (left) and 6 (center) versus PA domain 4 (right).

The structure of Vpb4Da2 was solved by X-ray crystallography to 3.2 Å resolution in the space group *C2221* with one molecule in the asymmetric unit. The Vpb4Da2 structure (Fig 1A) depicts an overall architecture of six structural domains. Domain 1 (amino acids 1–263), which encompasses a characteristic PA14 domain, adopts a conformation of ten anti-parallel β-strands, six small α-helices, and binds two adjacent calcium ions visible in the structure. Domain 2 (amino acids 264–483) consists of a β-hairpin motif, seven anti-parallel β-strands, a partially structured long loop, and three additional α-helices. Domain 3 (amino acids 484–593) has a β-sandwich fold of seven β-strands and four α-helices. Domain 4 (amino acids 594–730) consists of β-strands exhibiting a classical β-jelly roll fold and harboring a calcium ion. Domain 5 (amino acids 731–820) has an immunoglobulin-like fold of six β-strands and one α-helix. Domain 6 (amino acids 821–937), like domain 5, adopts an immunoglobulin-like fold with seven β-strands and two α-helices.

Vpb4Da2 exhibits an overall β-strand-rich structure and shares structural homology to PA [16], namely the domain conformation and organization spanning approximately the first 600 amino acids that include domains 1–3 (Fig 1A). Using the Superpose Superimposition (SSM) utility in the Coot program [29], structural alignment of domains 1–3 from PA with those of Vpb4Da2 determines a root-mean-square-deviation (rmsd) of the alpha-carbon (C_α_) of 2.69 Å. Single domain analysis using InterPro [42] designates Vpb4Da2 domain 4 as a galactose-binding-like domain (IPR008979) exhibiting distinct topology (Fig 1B) and conformation (Fig 1C) from the receptor binding domain 4 of PA (C_α_ rmsd = 4.87 Å). Additionally, Vpb4Da2 domain 4 adopts a conformation that resembles domain 3 of *Bt* Cry3Bb1 (PDB ID 1ji6) [43] and the *Clostridium thermocellum* xylanase carbohydrate binding module (PDB ID 1gmm) [44] (Fig 1C). The Vpb4Da2 distal carboxyl terminal domain 6 displays structural similarity to domain 4 of PA (C_α_ rmsd = 2.24 Å), (Fig 1D).

**Fig 2:**
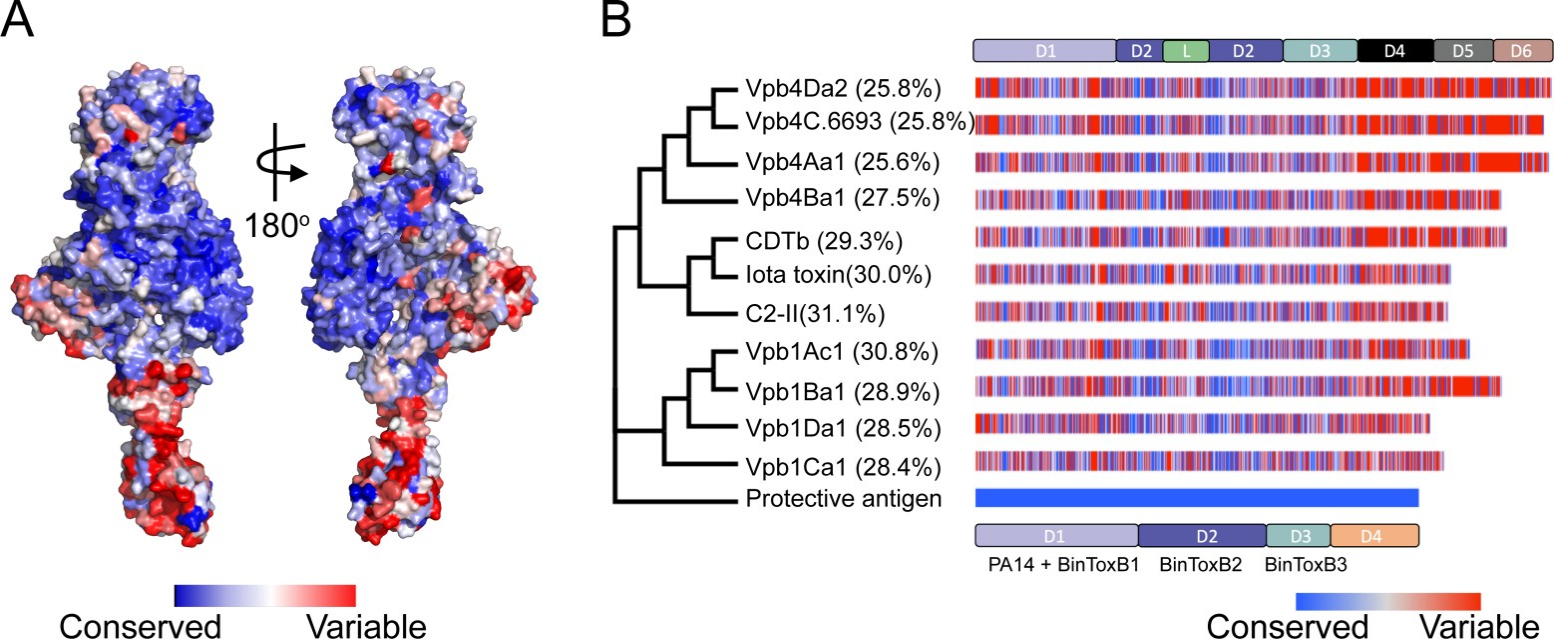
Structural conservation of Vpb4Da2 domains and its evolutionary relationship to Bacterial_exotoxin_B homologs. **(A)** Mapping amino acid sequence conservations and variabilities from multiple sequence alignments of Vpb4Da2 homologs onto the surface structure of Vpb4Da2. The degree of conservation is color-coded (high conservation = blue; low conservation = red). Higher sequence variability occurs within domains 4–6, whereas more sequence conservation is found in domains 1–3. **(B)** Phylogenetic tree of relative relationships between Bacterial_exotoxin_B homologs. Tip annotations show protein and global percent identity relative to PA precursor (AAA22637.1). Sequences used in this analysis include Vpb4Da2 (AZJ95709.1), Vpb4C.6693 (this study), Vpb4Aa1 (AEB52299.1), Vpb4Ba1 (OUB778819.1), CDTb (AUA37847.1), Iota toxin component Ib (CAA51960.1), C2 toxin component-II (BAA32537.1), Vpb1Ac1 (AEH05932.1), Vpb1Ba1 (AAR40886.1), Vpb1Ca1 (AAO86514.1), Vpb1Da1 (CAI40767.1), and Protective antigen precursor (AAA22637.1).

Evolutionary conservation and diversity of amino acids within protein modules inform on their functional importance. The Vpb4Da2 primary amino acid sequence was used to query the *Consurf* [36] server and the degree of evolutionary conservation for each amino acid residue determined. When the determined conservations were transposed onto the structural surface of Vpb4Da2, significant sequence variability was observed in domains 4–6, whereas domains 1–3 had high sequence conservation (Fig 2A). Additionally, the phylogenetic relationship between Vpb4Da2 and selected homologs, including CDTb, C-II component, Iota toxin component-Ib, PA, Vpb1s, and Vpb4s (Fig 2B) was examined. Vpb4Da2 clusters with closely related Vpb4Aa1, Vpb4Ba1, and Vpb4C.6693 (non-larvicidal to WCR, S3 Table) and is a distant homolog to CDTb, C2-II component, Iota toxin component-Ib, Vpb1s, and PA.

### Comparison of Vpb4Da2 structure with that of protective antigen (PA)

**Fig 3:**
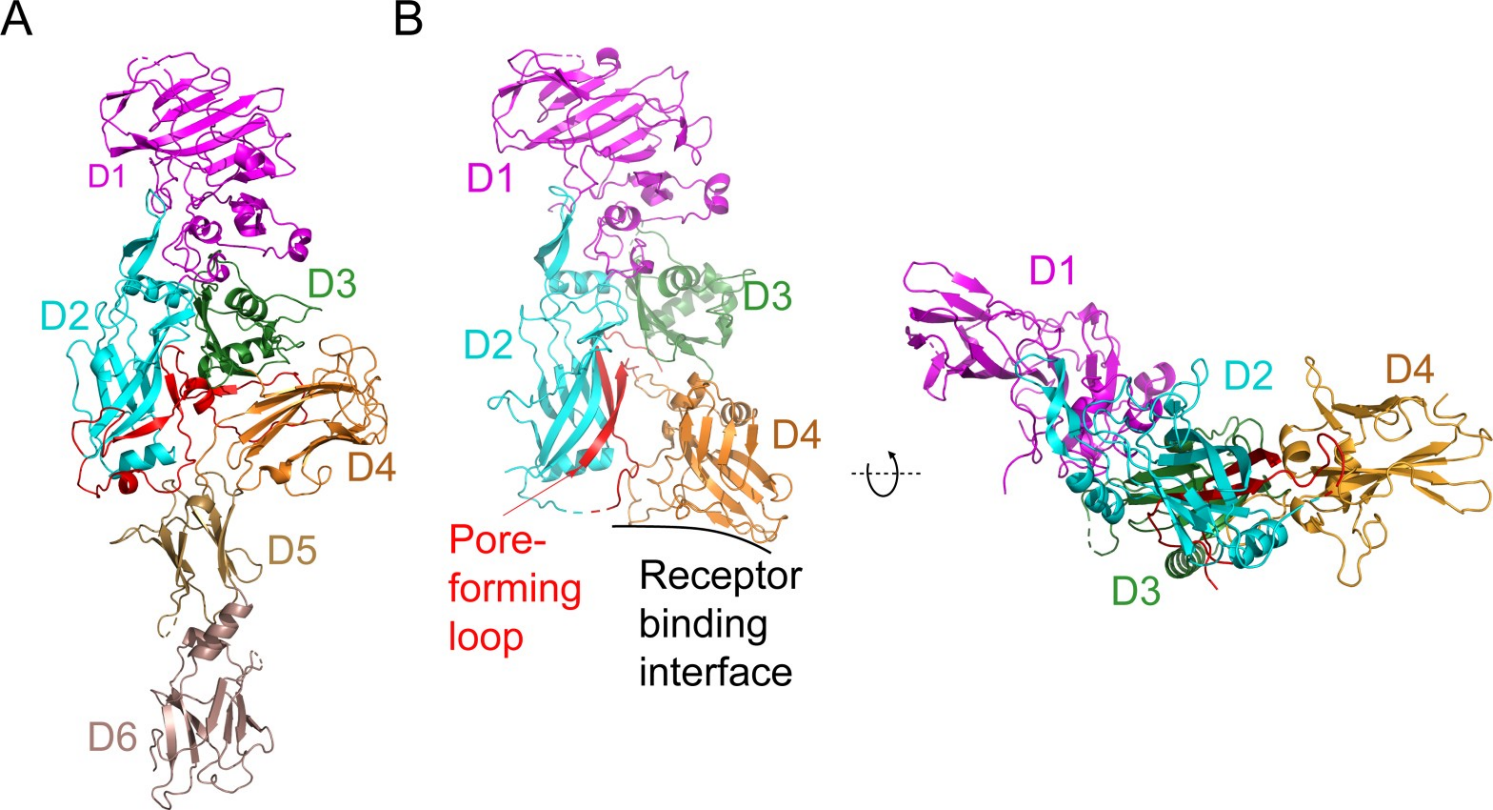
Structural comparison of Vpb4Da2 domains to corresponding domains of the Protective Antigen (PA). **(A)** Unique to Vpb4Da2, when compared to PA (PDB ID Acc1), are additional domains 5–6 found at the carboxyl-terminus. Moreover, Vpb4Da2 domain 4 displays a distinct topology. **(B)** Domains 1–3 of both proteins exhibit similar structural organization. The receptor binding interface and pore forming loop of PA are indicated.

Herein, we describe for the first time the three-dimensional structure of a Vpb4 class of protein (Vpb4Da2) exhibiting insecticidal activity against WCR. Structural and functional analyses indicate that organizationally, the amino-terminal half of Vpb4Da2 is shared among members of the Bacterial_exotoxin_B family (Fig 3A and 3B). The first half of the Vpb4Da2 protein contains a domain 1 where the proteolytic site is found to promote oligomerization, a domain 2 comprising the suggested pore-forming stem region, and a domain 3 involved in oligomerization [22]. Unique to Vpb4Da2 is its domain organization at the carboxyl-terminal end of the protein (Fig 3A). While three carbohydrate binding modules (CBM) are found in the carboxyl-terminal end of Vpb4Da2, only one and two CBMs have been described respectively for the carboxyl-terminus of PA (Fig 3B) [18] and for the *Clostridium difficile* CDTb [45].

### Vpb4Da2 is proteolytically-processed to a stable core

**Fig 4:**
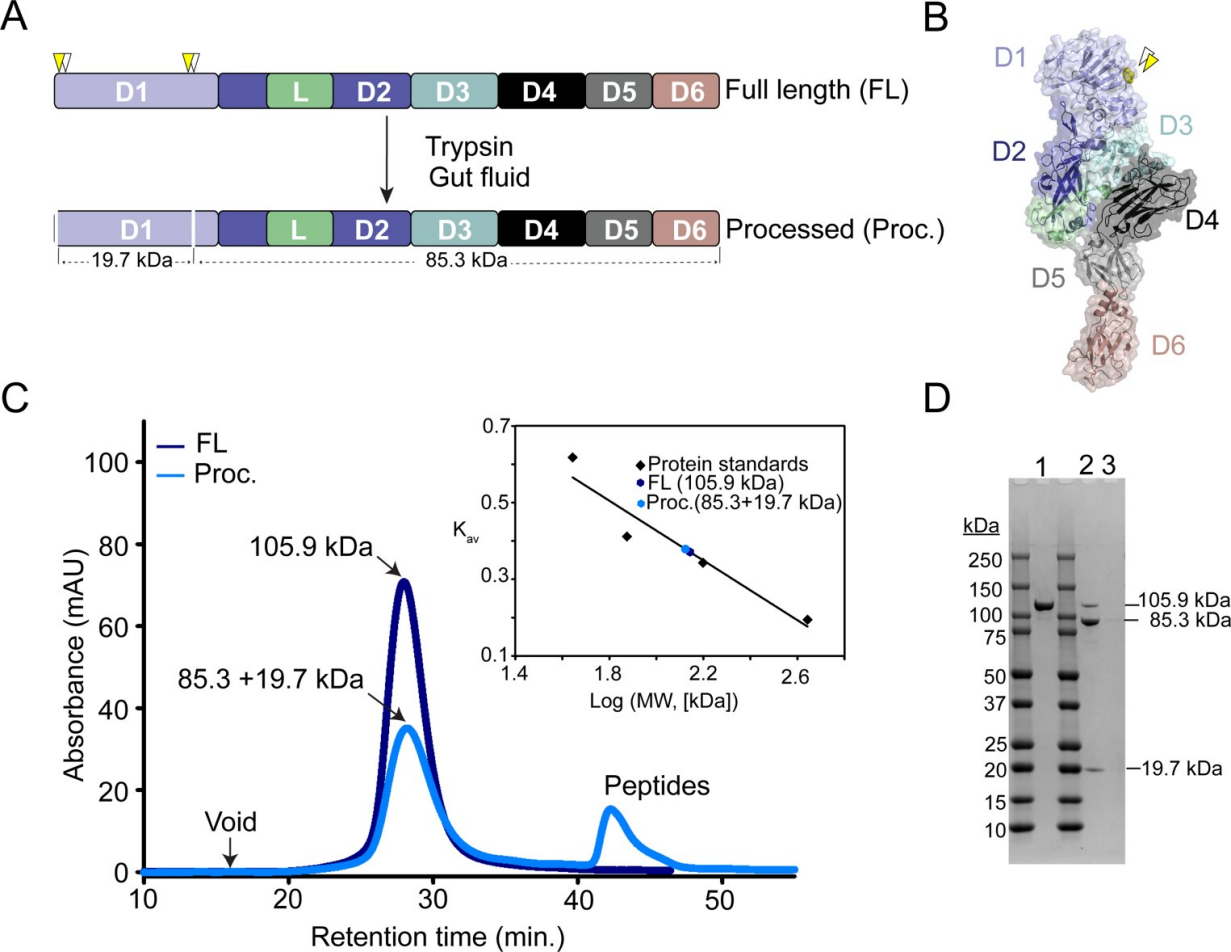
Vpb4Da2 processing by proteases occurs within domain 1. **(A)** Schematic representation of Vpb4Da2 domains and protease cleavage sites. Yellow and white arrowheads are gut fluid and trypsin cleavage sites, respectively. Cleavage sites at the amino terminal end, within domain 1 are indicated by clear vertical lines. **(B)** Surface rendition of Vpb4Da2 structure showing proteolytic cleavage sites (yellow spheres). **(C)** Analytical size-exclusion chromatography (SEC) profiles of the full-length (dark blue trace) and the trypsin-processed (light blue trace) Vpb4Da2. Full-length (105.9 kDa) and processed (85.3 kDa and 19.7 kDa complex) protein samples exhibit approximately the same retention time when ran separately through an SEC column. The calibration curve is shown in the inset and the experimentally determined molecular weights of full-length and processed Vpb4Da2 are indicted by dark blue and light blue squares, respectively. **(D)** The SDS-PAGE profile of the elution peaks from the full-length Vpb4Da2 (lane 1) and processed (lane 2) samples, along with the molecular size of the eluted protein bands, are shown. The SDS-PAGE profile of the elution peak at approximately 42 min designated “Peptides” in (C) and derived from processed Vpb4Da2 ran through the SEC column is shown in lane 3. No apparent peptides of molecular weights higher than 10 kDa were observed (lane 3), suggesting that peptides are very small breakdown products of the proteolytic reaction.

To assess whether Vpb4Da2 is processed and forms a stable core in the WCR midgut, as found in other proteins active against WCR, extracts from neonates fed for 24 h with fluorescently-labeled Vpb4Da2 were analyzed using SDS-PAGE. Vpb4Da2 forms a stable core in the WCR midgut represented by a protein band of approximately 80 kDa (S1A Fig). Furthermore, *in vitro* proteolytic processing with WCR gut fluid extract maps the cleavage site at amino acid position 181 in domain 1. Sporadically, we observed cleavage by gut fluid at the distal carboxyl-terminal end of domain 6 within the last 9 amino acids. The significance of this observation is unclear. Moreover, *in vitro* processing of Vpb4Da2 by trypsin identified a cleavage site at amino acid position 179 in domain 1, near the WCR gut fluid cleavage site (Fig 4A and S2 Fig), indicating a solvent-exposed flexible region within domain 1 prone to proteolytic processing. Diet bioassays demonstrate that the larvicidal activity against WCR of the trypsin-processed Vpb4Da2 is equivalent to that of the wild-type protein (S2 Table). Additionally, trypsin-processed and wild-type Vpb4Da2 migrated at an equivalent volume when injected onto a size-exclusion column (Fig 4C), indicating that the trypsin-processed Vpb4Da2 fragment remains attached to the rest of the molecule.

### Vpb4Da2 domains 4, 5, and 6 contribute to activity against WCR

**Fig 5:**
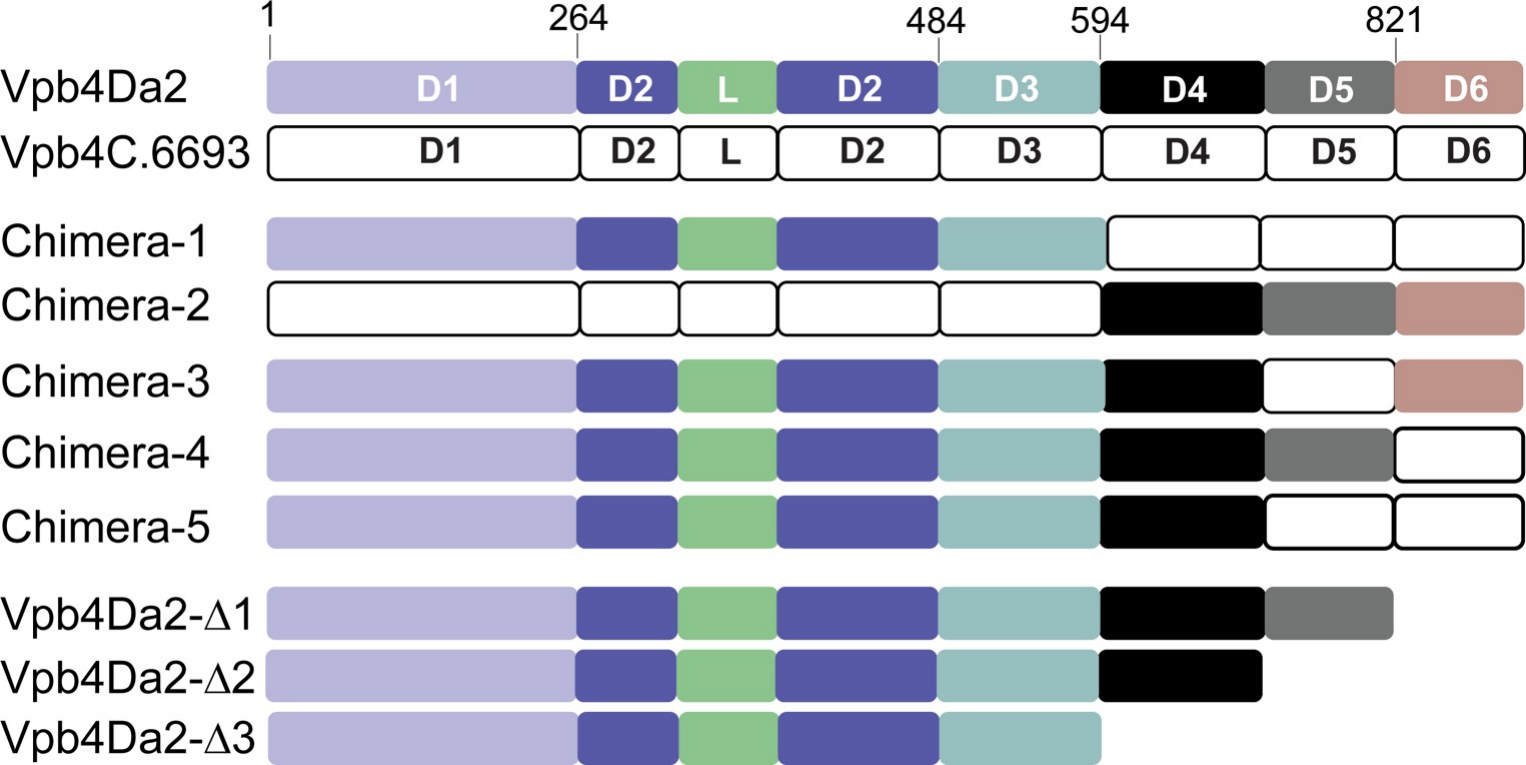
Schematic representation of Vpb4Da2 and Vpb4C.6693 domain-swap and truncation variants. Domains and putative pore-forming loop (L) are labeled and color coded.

To determine whether insecticidal activity is mediated by the Vpb4Da2 carboxyl-terminal domains, truncated Vpb4Da2 and domain-swap variants were engineered and assessed in bioassays. Domain-swaps were conducted between Vpb4Da2 and Vpb4C.6693 (S3 Fig). Domain-swap (chimeras -1, -2, -3, -4, and -5) and truncated (Vpb4Da2-Δ1, -Δ2, and -Δ3) variants are depicted in Fig 5. Using thermal stability assessment, most chimeric variants exhibited melting temperatures (*T*_m_) close to wild-type Vpb4Da2 (50°C), (Table 1), suggesting that their conformational stability was not significantly altered.

**Table 1:** Insecticidal activity of Vpb4Da2, Vpb4C.6693, and corresponding chimeras and deletions.

Vpb4Da2 and variants	Mean % mortality at 44.12 μg/cm^2^ (± SD)^a^	*T*_*m*_ (± SE)
Vpb4Da2	100.00 ± 0.00 *	50.00 ± 0.00
Vpb4C.6693	3.57 ± 7.15	54.00 ± 0.19
Chimera-1	0.00 ± 0.00	54.75 ± 0.08
Chimera-2	91.43 ± 10.17 *	48.58 ± 2.17
Chimera-3	23.81 ± 5.05 *	52.50 ± 0.33
Chimera-4	22.32 ± 21.10	49.58 ± 0.08
Chimera-5	18.75 ± 14.23	54.17± 0.50
Vpb4Da2-Δ1	7.14 ± 14.29	N/A
Vpb4Da2-Δ2	3.57 ± 7.15	N/A
Vpb4Da2-Δ3	10.72 ± 13.68	N/A

^a^ Means followed by asterisks are significantly different from buffer control treatment at *p*-value < 0.040. Dunnett’s test was used. Melting temperature (T_m_).

Purified chimeric proteins were used to assess larvicidal activity against WCR. Of the five chimeric proteins evaluated, chimera-2, a Vpb4C.6693 domain 4–6 swapped with the corresponding domains from Vpb4Da2 (amino acid 594 to 937) exhibited 91.43% mortality in comparison to wild-type Vpb4Da2 (Table 1). Moreover, a Vpb4Da2 chimera in which domain 5 was swapped with the equivalent domain 5 from Vpb4C.6693 (chimera-3) retained partial insecticidal activity (Table 1 and S3 Table). All remaining chimeras (chimeras -1, -4 and -5) had no larvicidal activity when evaluated against WCR (Table 1 and S3 Table). Further assessment with truncated variants indicated that a loss of larval toxicity occurred when the Vpb4Da2 carboxyl-terminal domains 4 to 6 were truncated (Fig 5, Table 1, and S3 Table), suggesting that these domains are essential for Vpb4Da2 insecticidal activity against WCR.

### Amino acid substitution on the Vpb4Da2 pore-forming module affects WCR toxicity

**Fig 6:**
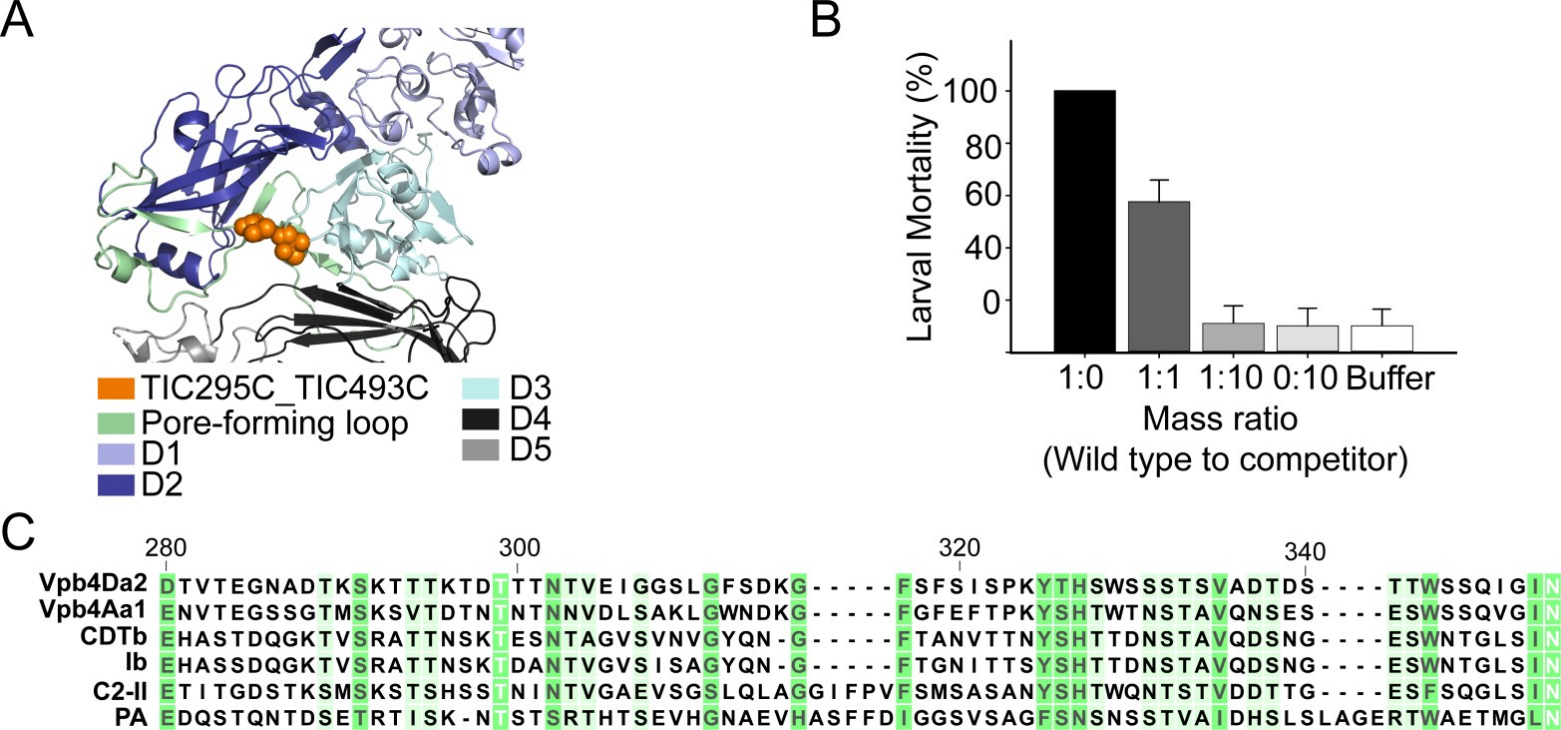
Competition bioassay evaluation using the pore-forming disabled variant. **(A)** Structural representation (PyMOL™2.0.2) of the Vpb4Da2 pore-forming loop within domain 2 (pale green) and amino acid substitutions (orange spheres) representing the disabled insecticidal protein (DIP) variant. Domains D1-D5 are also represented. **(B)** Mass action *in vivo* competition bioassay. Wild-type Vpb4Da2 was competed with increasing challenge ratio of the Vpb4Da2-DIP variant. Bars represent the mean percent larval mortality with standard error. Mean values with same letter are not statistically different (One Way ANOVA Student-Newman-Keuls’ test, α = 0.05). **(C)** Multiple sequence alignment of the Vpb4Da2 pore-forming loop and selected β−PFPs from the Bacterial_exotoxin_B family using CLCBio™ version 7.6.4. Numbers on sequences are relative to Vpb4Da2. The degree of sequence conservation is represented by a green background gradient.

To assess whether the Vpb4Da2 domain 2 extended loop (amino acids 280 to 354) functions as a putative pore forming module, cysteine substitutions were introduced to allow chemical cross-linking or disulfide bond formation. Of several double-cysteine variants screened for loss of insecticidal activity due to putative deficient pore-forming properties, variant Vpb4Da2-T295C_T493C (Fig 6A) was selected as a disabled insecticidal protein (DIP) [32]. Mass action competition bioassay analysis was performed as described by Jerga *et al*. [32]. The Vpb4Da2-DIP variant, being receptor-binding competent but non-insecticidal due to loss of the putative pore-forming property, competes with the wild-type Vpb4Da2 insecticidal protein for activity (Fig 6B).

### Brush border membrane bound Vpb4Da2 forms a pH dependent oligomer

**Fig 7:**
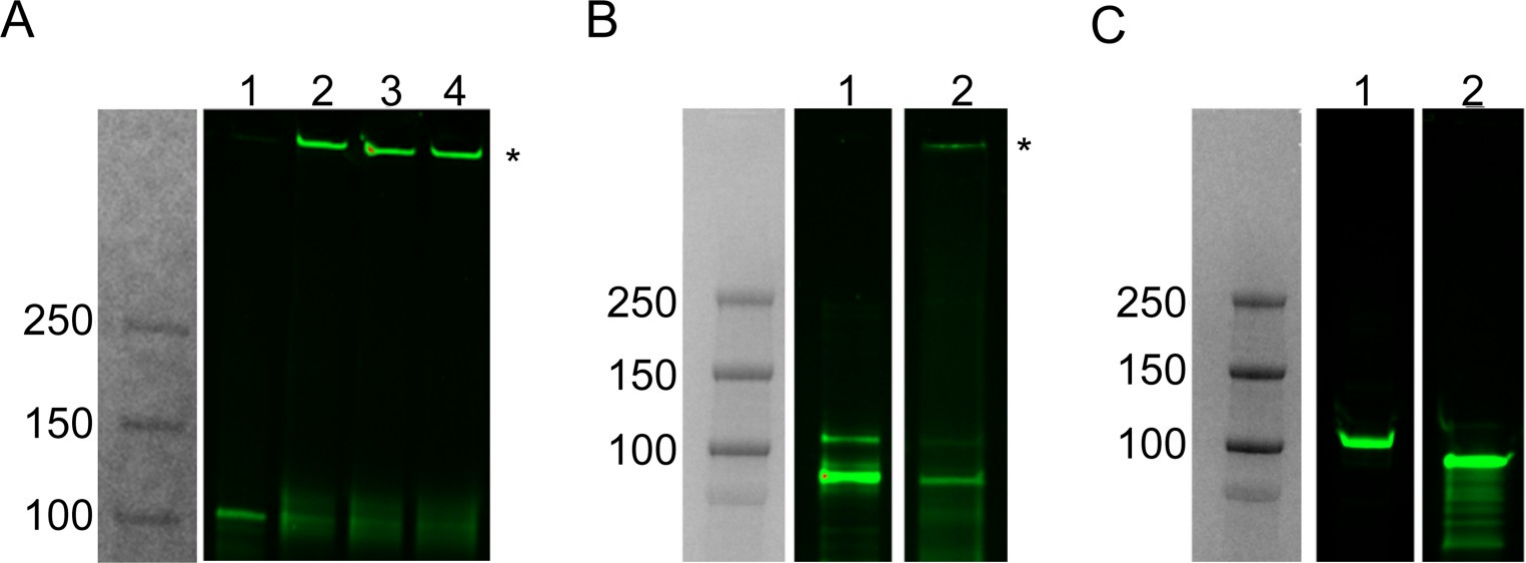
Vpb4Da2 forms an oligomer at acidic pH in the presence of the BBM from WCR (Full SDS-PAGE figures are depicted in supporting information). **(A)** Trypsin-processed Vpb4Da2 (tryptic core) forms a reversible oligomer in the presence of WCR BBM as shown in the gel following treatment with a homo-bifunctional cross-linker (BS3). BS3 titration at 0, 1, 2, and 5 mM are in lanes 1, 2, 3, and 4, respectively. Reactions were carried out at pH 6.0 and at room temperature. The oligomerized Vpb4Da2 tryptic core is indicated by the asterisk at the entrance of gel wells. **(B)** The Vpb4Da2 tryptic core forms a BBM bound oligomer in the absence of crosslinker at pH 6.0 and 37°C. The Vpb4Da2 tryptic core without BBM is shown as a monomer (lane 1). An SDS-resistant oligomer, asterisk (lane 2), is formed in the presence of WCR BBM. **(C)** The trypsin-processed Vpb4Da2 disabled insecticidal protein (DIP) variant does not form an SDS-resistant oligomer in the presence of WCR BBM. The full-length Vpb4Da2 and the processed DIP variant are in lanes 1 and 2, respectively. All reactions were at pH 6 and at 37°C.

To evaluate whether Vpb4Da2 forms an oligomer as observed in other *Bt* proteins, the fluorescently labeled and insecticidal Vpb4Da2 variant, Vpb4Da2-K733C_A422C, was analyzed in the presence of WCR BBM harboring putative receptors. Initial demonstration that the trypsin-processed Vpb4Da2 forms a dose-dependent oligomer at an optimum pH 6.0 in the presence of the homo-bifunctional crosslinker (BS3) was performed (Fig 7A). In a subsequent assessment, a fraction of the trypsin-processed Vpb4Da2 was shown to accumulate as an SDS-resistant oligomer at the top of the gel, due to its anticipated higher molecular weight in the presence of WCR BBM, at pH 6.0 and at 37°C without crosslinker (Fig 7B). Conversely, the unprocessed wild-type Vpb4Da2 and the full-length pore-forming DIP variant did not form an SDS-resistant oligomer under the same conditions (Fig 7C). This result indicates that the trypsin-processed Vpb4Da2 variant exhibits an oligomeric state under acidic conditions characteristic of the WCR midgut environment [46].

### Vpb4Da2 effects on WCR larvae and midgut cells and tissue

**Fig 8:**
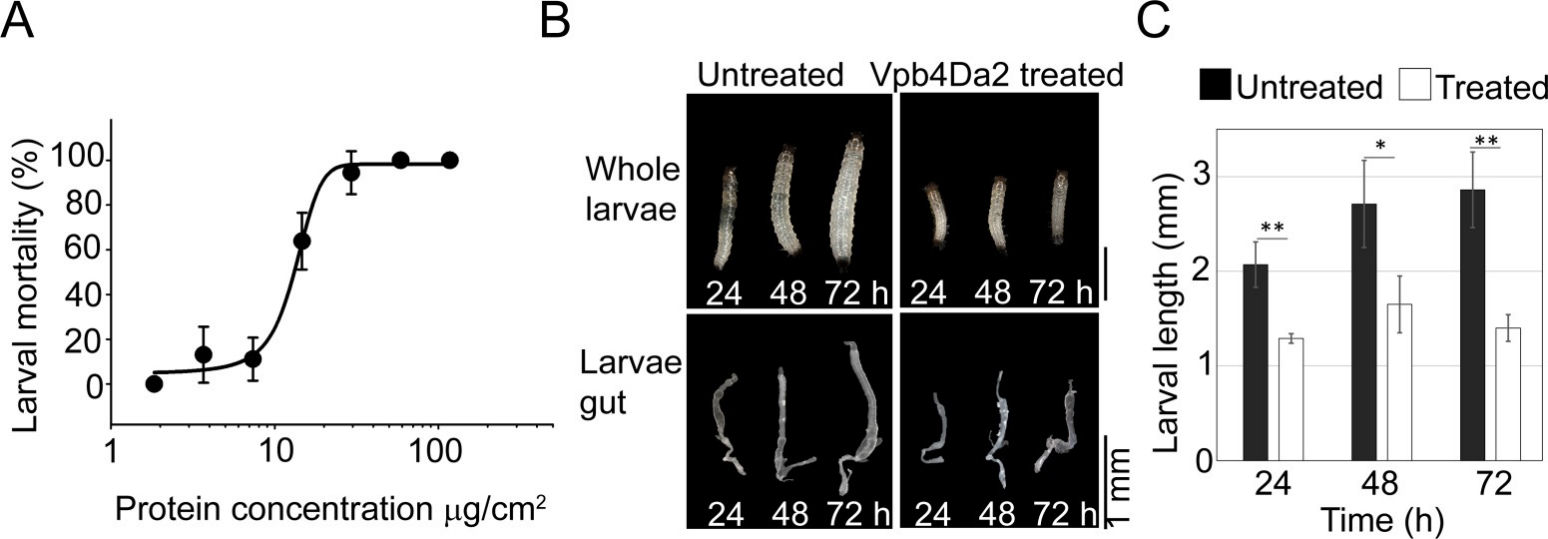
Effects of Vpb4Da2 on WCR larvae development and dissected gut morphology. (A) Vpb4Da2 larvicidal activity on WCR measured by increasing protein concentration. Data points represent mean mortality (± SD), not corrected for buffer control mortality (5.56%). (B) Larval growth inhibition and gut morphology evaluation from 24 h to 72 h following exposure to Vpb4Da2. Larval growth was inhibited as early as 24 h. Dissected midguts from treated and untreated larvae are also shown. (C) Bar graph representation of growth inhibition. Data represent mean larval length (± SD). Asterisks indicate statistical significance at p = 0.05 (*) and p = 0.01(**) using the student-t test (n = 5).

**Fig 9:**
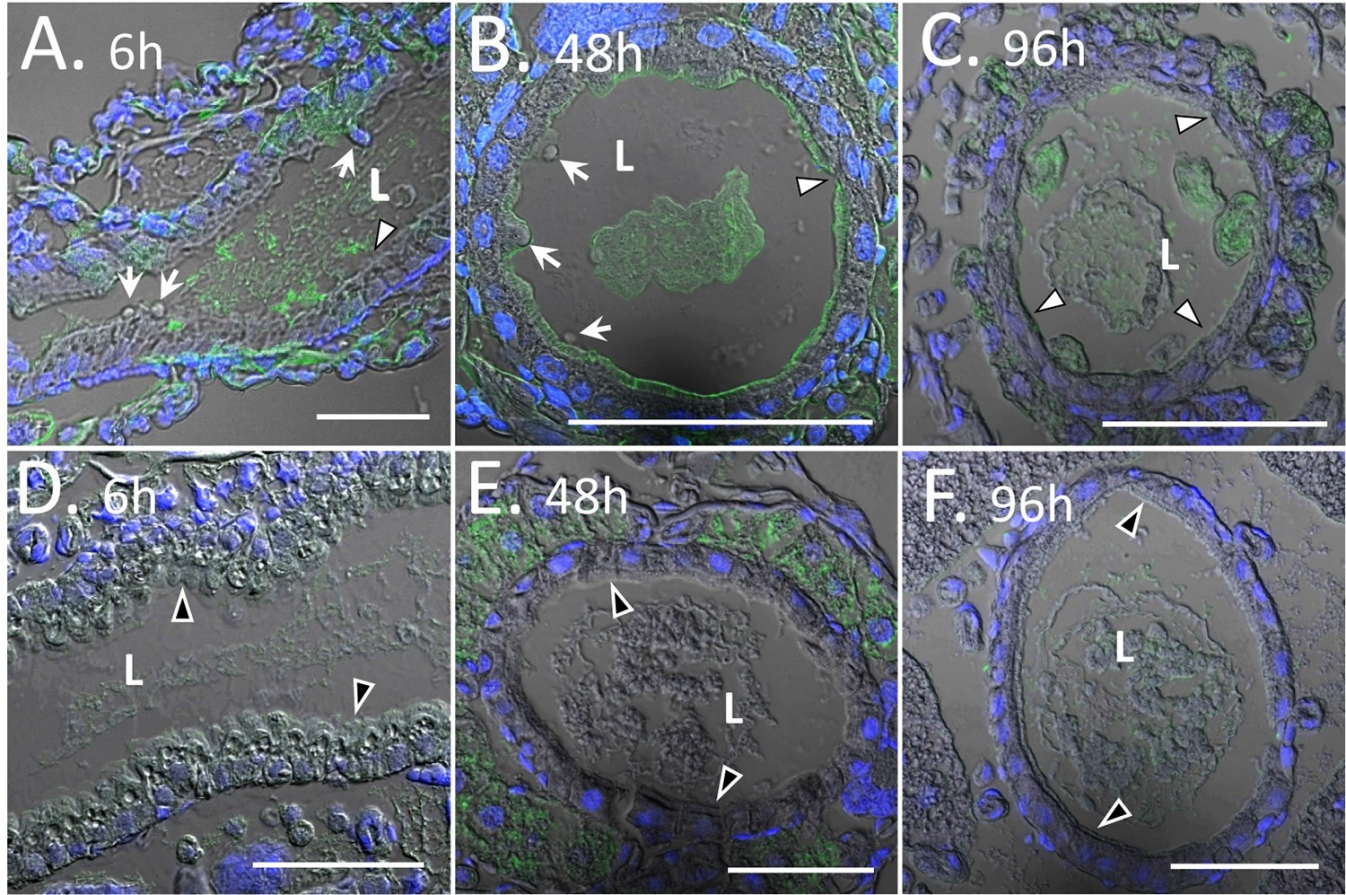
Western corn rootworm midgut morphology after feeding with Vpb4Da2. Vpb4Da2 staining by Alexa-fluor-488 conjugated secondary antibody is in green. Nuclei staining by DAPI is in blue. **(A)** Longitudinal section of the midgut after 6 h exposure to Vpb4Da2. **(B)** Cross section after 48 h exposure to Vpb4Da2. **(C)** Cross section after 96 h exposure to Vpb4Da2. (A) and (B) White arrows indicate blebbing of the epithelial cells. (A), (B) and (C) White arrowheads designate a complete or near complete loss of the apical microvilli layer. **(D)**, **(E)**, and **(F)** Are untreated controls where black arrowheads indicate the intact apical microvilli layer. Scale bars are all 50 μm.

WCR growth inhibition was observed as early as 24 h post-exposure to Vpb4Da2 (Fig 8B and 8C). Cross-sectional and longitudinal analyses of WCR neonates fed with Vpb4Da2 were conducted to evaluate its effects on the insect midgut cells and tissue. Following ingestion, Vpb4Da2 promoted blebbing of the epithelial cells as early as 6 h (Fig 9A). At 48 h of exposure, continuous blebbing of the epithelial cells and Vpb4Da2 binding to the apical microvilli along with sloughing off cellular debris in the WCR lumen were observed (Fig 9B), as well as tissue damage manifested by a partial disruption of the apical microvilli layer. Extensive damage occurred 96 h post Vpb4Da2 ingestion, revealing a near complete loss of the microvilli layer, (Fig 9C).

## Discussion

In the current study, we characterized Vpb4Da2 a newly identified *Bt* β-PFP with insecticidal activity against WCR. We described the crystal structure of Vpb4Da2, determined its putative receptor binding domains responsible for activity against WCR, and determined that its underlying mode of action (MOA) mirrors that of the familiar, well-characterized 3-domain *Bt* proteins. Analysis of the Vpb4Da2 structure determined by X-ray crystallography revealed a protein rich in β-strands exhibiting a six-domain architecture. Vpb4Da2 has an overall structural homology to the Bacterial_exotoxin_B family of the β-barrel pore-forming proteins (β-PFPs) [21, 47, 48], namely the structural region spanning domains 1–3 of the *B*. *anthracis* PA protein [16] and that of the *C*. *botulinum* C2-II component [17]. The notable structural (Fig 1) and sequence (Fig 2) variations at the C-terminus between Vpb4Da2 and these structural homologs, suggest that the C-terminus of Vpb4Da2 is involved with putative receptor binding. This is not without precedent as receptor recognition and domain-based specificity were also reported for other rootworm-active proteins [7]. Walters et *al*. [7] replaced domain 3 of the *Leptinotarsa decemlineata*-active Cry3Aa with that of Cry1Ab. The resulting hybrid protein, eCry3.1Ab, exhibited increased insecticidal activity against WCR conferred by the variable-region of the Cry1Ab domain 3. Domains 2 and 3 of three-domain *Bt* Cry proteins mediate receptor recognition and insecticidal specificity [49–52]. De Maagd *et al*. [53] reported a hybrid protein with domains 1 and 2 of Cry1Ab and domain 3 of Cry1C exhibiting different brush border membrane protein binding compared to wild-type proteins with higher insecticidal activity against *Spodoptera exigua*. The authors further demonstrated the implication of domain 3 in binding to a 200 kDa putative receptor.

Given that putative receptor recognition is most likely key to the insecticidal activity of Vpb4Da2, and that domain 4 of PA was reported to be part of its receptor binding region, we wanted to assess the function of domain 4 and the C-terminal extension (domains 5 and 6) of Vpb4Da2. We took advantage of Vpb4C.6693, a Vpb4 protein with 77% overall amino acid sequence identity to Vpb4Da2 but inactive against WCR and conducted a carboxyl-terminal domain swap analysis. The hybrid variant, Vpb4C.6693-Vpb4Da2 (chimera-2), with domains 1 through 3 of Vpb4C.6693 and domains 4 through 6 of Vpb4Da2, displayed WCR larval susceptibility comparable to that of the wild-type Vpb4Da2. However, the reciprocal hybrid, Vpb4Da2-Vpb4C.6693 (chimera-1), exhibited complete loss of insecticidal activity. This observation clearly indicates that domains 4–6 of Vpb4Da2 are required for toxicity, and given structural homology to known receptor binding domains, this observation suggests that receptor binding is conferred by unknown solvent-exposed residues in domains 4–6. Further analysis to determine the extent of the binding contribution from each individual domain demonstrated that loss of WCR insecticidal activity was associated with swapping Vpb4Da2’s domain 5 and domain 6 out individually, or in tandem, suggesting their integral role in putative receptor binding and insecticidal activity. Another key aspect of Vpb4Da2’s unique domain 5 and 6 C-terminal extension is that they connect to domain 4 at a structurally identical position where PA has its receptor binding epitope (Fig 3). Given this steric occlusion, the Vpb4Da2 structure suggests that it has a different receptor recognition site than PA, likely comprised by its domains 5 and 6 or domains 4 through 6 (Fig 3). Furthermore, unlike the PA domain 4 described as a receptor binding domain [54] with an immunoglobulin-like fold [16], Vpb4Da2 domain 4 adopts a fold of the carbohydrate binding module 6 (CBM6) [44], which is also found in domain 3 of many three-domain Cry proteins [55]. This difference in PA and Vpb4Da2 domain 4 provides additional supporting evidence of a divergent receptor binding partner(s).

Binding domain duplication within a specific lectin has been reported to increase cognate receptor binding affinity through avidity effects [56, 57]. Additionally, Voordeckers *et al*. [58] described heterogeneous clustering of carbohydrate binding modules as a gene duplication event providing the basis for new functionalization. The putative receptor binding region of Vpb4Da2 displays a cluster of one distinct module (domain 4) and a duplicated module (domains 5 and 6). It is therefore conceivable that domain duplication along with a possible domain 4 insertion had provided a heterogeneous clustering which evolutionarily might have arisen to confer the Vpb4Da2 receptor binding property.

Besides determining that Vpb4Da2’s unique C-terminal domains 4–6 were required for WCR activity, another key question we wanted to address was whether these alone were sufficient for WCR activity. Herskowitz [59] introduced the dominant negative concept and defined that “a dominant negative mutant protein will retain an intact functional subset of the domains of the parent, wild-type protein, but have the complement of this subset either missing or altered so as to be non-functional”. Herskowitz also indicated that a monomeric protein deficient in oligomerization can also be inhibitory if there is limiting amount of substrate. Bt receptors, which are key in conferring the spectrum of insecticidal activity to three-domain Cry proteins, are displayed on the midgut epithelium and are generally less abundant than the insecticidal proteins used in these assays. Thus, a monomeric disabled variant that is deficient in the putative pore-formation function, but otherwise has an unaltered receptor binding domain(s) would compete against the native counterpart on a target insect if the disabled variant is mixed in large excess with the native wild-type protein. Vpb4Da2-T295C_T493C was effective in competing away the wild-type protein’s insecticidal activity. The Vpb4Da2-T295C_T493C disabled variant has modifications in the stem region in domain 2, which has been described previously as a conserved structural motif for all Bacterial_exotoxin_B proteins and deemed critical to form the extended heptameric β-barrel oligomer, but otherwise having an unaltered Domains 4–6 C-terminus. Thus, we deduced that Vpb4Da2-T295C_T493C was unable to form pores but could compete with the wild-type protein using its intact receptor binding domain. Our conclusion is the function of Vpb4Da2’s domains 4–6 is required but is not sufficient for WCR insecticidal activity.

Insecticidal protein processing by insect gut proteases is integral to the well-known insecticidal MOA leading to target insect mortality [60–62] and is often described as an activation phase manifested by an active stable core formation. This initial step has been demonstrated for proteins belonging to the 3D-Cry [63], ETX_MTX2 [31], Toxin_10 [9], and Vip3 [64] protein classes. In the current investigation, Vpb4Da2 was shown to be processed by WCR gut proteases. Using WCR gut fluid extract and commercial trypsin, cleavage sites were mapped to the same solvent-exposed loop within domain 1. Subsequent size-exclusion chromatography demonstrated that protease-processed Vpb4Da2 comprised a cleaved portion of domain 1, which remained non-covalently attached to the rest of the protein. This is not without precedent, as Yamaguchi T *et al*. [65] described a nick in the loop between the secondary structure alpha-3 and alpha-4 of Cry8Da domain 1 as critical for binding to specific proteins on the BBM of the Japanese beetle, *Popillia japonica* Newman. Proteolytically-processed Vpb4Da2 insecticidal activity against WCR was found to be similar to wild-type. Conversely, protease processing of Vpb4Da2 can promote oligomer formation at acidic pH in the presence of BBM (Fig 7). It is therefore possible that the observed oligomer is a precursor to a pore complex. It is also important to note that both full-length and proteolytically-processed Vpb4Da2 demonstrate insecticidal activity against WCR indicating the proteolytically-processed moiety of Vpb4Da2 domain 1 does not recruit an enzymatic component as described for PA [66] and other Bacterial_exotoxin_B proteins [15, 18].

All Bacterial_exotoxin_B proteins reported thus far exhibit a conserved phenylalanine residue in their specific pore lumen, described as a ϕ-clamp, modulating pore formation and translocation of cognate enzymatic components into the cytosol [47, 67, 68]. In PA and C2-II, substitution of the ϕ-clamp phenylalanine with alanine resulted in loss of the B-component aptitude to translocate protein [68, 69]. Interestingly, amino acid sequence comparisons reveals the absence of the conserved phenylalanine in Vpb4Da2 which is present in PA and C2-II (S2 Fig) and critical for threading the enzymatic component of PA [70] and C2-II [69] into the cytosol. Instead, Vpb4Da2 has an alanine at that same amino acid position, suggesting that Vpb4Da2 is likely unable to perform polypeptide translocation, and its activity against WCR does not require any enzymatic partner. Indeed, purified Vpb4Da2 is highly active in WCR bioassays, suggesting that additional partners are not required.

Histopathology and immunohistochemistry analyses of the midgut from Vpb4Da2-fed WCR further highlight binding as an essential step in the MOA of Vpb4Da2. As early as 24 h post-exposure, susceptible larvae stopped growing, suggesting an early onset of Vpb4Da2 insecticidal activity. Along with binding to WCR midgut apical microvilli, ingested Vpb4Da2 promotes epithelial cell blebbing and cellular debris shedding in the insect lumen. Extensive loss of microvilli was observed with continued exposure to Vpb4Da2. Blebbing and shedding of micro-vesicles have been regarded as a repair mechanism to protect cellular plasma membrane against damage from pore-forming proteins [71]. Therefore, it is conceivable that the blebbing and shedding observed when WCR was intoxicated with Vpb4Da2 were the result of plasma membrane injury repair from pore formation. Consistent with this repair mechanism, cellular debris expelled from *Caenorhabditis elegans* intestinal microvilli into the lumen in response to intoxication by the pore-forming protein, Cry5B, was described as a pore removal mechanism to restore the integrity of injured plasma membrane [72]. A similar mechanism was also found for the cholesterol-dependent cytolysins (CDCs) [73]. Additionally, the appearance of blebs and large vesicles has also been described for WCR gut epithelium exposed to the Gpp34Ab1/Tpp35Ab1, Cry3Aa1, and Cry6Aa1 proteins [74, 75], suggesting commonality with Vpb4Da2 histopathology toward WCR.

In conclusion, X-ray crystallography, biochemical analyses, and immunohistochemistry were used to elucidate the MOA of Vpb4Da2 a *Bt* protein exhibiting insecticidal activity against WCR.

## Supporting information

S1 Table Structure solution and refinement parameters. (DOCX)

S2 Table Susceptibility of WCR larvae to Vpb4Da2 and derivatives used in the current study. ^a^ Total number of insects tested per dose. ^b^ Means followed by an asterisk are significantly different from untreated control at *p*_value < 0.04.^c^ Tryptic core (trypsin processed) Vip4Da2.
(DOCX)

S3 Table Susceptibility of WCR larvae to Vpb4Da2, Vpb4C.6693, and derived chimeras and deletions. ^a^ Total number of insects used per dose. ^b^ Means followed by an asterisk are significantly different from untreated control at *p*_value < 0.04.
(DOCX)

S1 Fig Vpb4Da2 *in vivo* proteolytically-processed fragments exhibit a similar size as that found with *in vitro* WCR gut fluid processing. **(A)** *In vivo* fate of fluorescently labeled Vpb4Da2 after 24 h feeding of neonate WCR. Lane 1, labeled protein only; lane 2, WCR extract from buffer control treatment; lane 3, WCR extract from larvae treated with labeled Vpb4Da2. **(B)** *In vitro* WCR gut-fluid (GF) processing of Vpb4Da2 at pH 6.0. Lane 1 and lane 2 are un-processed full-length (solid arrow) and GF processed Vpb4Da2 (dash arrow), respectively. Protein band at ~10 kDa represents protease inhibitors.
(TIF)

S2 Fig Primary amino acid sequence alignment of Vpb4Da2, C2 component II, and protective antigen. Yellow arrowheads indicate WCR gut fluid cleavage sites while white arrowheads show trypsin cleavage sites. Domain boundaries are delineated in blue. Conserved phenylalanine, Φ-clamps, of C2-II and PA are in the red box. Numbers on sequences are relative to amino acid position 1. Sequence conservation gradient is from conserved (black shade) to diverse (no shade). Sequence alignment was performed using CLCBio™ version 7.6.4.
(TIF)

S3 Fig Primary amino acid sequence alignment of Vpb4Da2 and Vpb4C.6693. Alignment was obtained using CLCBio™ version 7.6.4. Sequence conservation and domain boundaries are also indicated.
(TIF)

S4 Fig Vpb4Da2 forms an oligomer at acidic pH in the presence of the brush border membrane from WCR (Full SDS-PAGE from Fig 7). (TIF)

S5 Fig Full validation report_7mjr. (PDF)

S1 File CLUSTAL W (1.81) multiple sequence alignment for analysis in Fig 2A. (TXT)

S2 File Amino acid sequence conservation score for analysis in Fig 2A. (TXT)

S3 File Size exclusion chromatography traces for Fig 4C. (XLSX)

S4 File Analysis of thermal stability for Table 1. (DOCX)

S5 File Mass action competition bioassay for Fig 6. ^a^ Means followed by asterisks are significantly different from buffer control treatment at *p*-value < 0.040. Dunnett’s test was used.
(DOCX)

S6 File Concentration dependent assessment of Vpb4Da2 insecticidal activity against WCR for Fig 8. ^a^ Means followed by asterisks are significantly different from buffer control treatment at *p*-value < 0.040. Dunnett’s test was used.
(DOCX)

S7 File Intact mass spectrometry analysis of Vpb4Da2 processed with trypsin (Fig 4). Molecular masses of proteolytically processed and full length Vpb4Da2 are shown. Fragment of 42,696 Da is the doubly charged form of the 85,391 Da fragment.
(TIF)

S8 File Intact mass spectrometry analysis of Vpb4Da2 processed with WCR gut fluid (Fig 4). Molecular masses of proteolytically processed and full length Vpb4Da2 are shown.
(TIF)

S9 File Amino acid sequence of Vpb4Da2 and Vpb4C.6693. (DOCX)
