# Supplementary material for: Structural and functional insights into the first Bacillus thuringiensis vegetative insecticidal protein of the Vpb4 fold, active against western corn rootworm
Source: PLoS One. 2021 Dec 20;16(12):e0260532. doi: 10.1371/journal.pone.0260532 (PMC8687597; doi:10.1371/journal.pone.0260532)
Supplement: S1 Table — (DOCX) [file pone.0260532.s001.docx]

| Space group | *C2221* |
| --- | --- |
| Unit cell lengths (Å) | a = 117.5, b = 221.4, c = 154.1 |
| Unit cell angles (°) | 90, 90, 90 |
| Resolution (Å) | 3.2-43.0 |
| No. of reflections | 228730 |
| Redundancy | 7.2 |
| <I/σ(I)> | 23.3 |
| Completeness (%) | 100% |
| R_merge-linear_ | 0.162 |
| R_merge-square_ | 0.115 |
| R_cryst_ | 0.196% |
| R_free_ | 0.241% |
| Protein residues/atoms | 899/14250 |
| Rmsd., bonds (A˚) | 0.011 |
| Rmsd., angles (°) | 1.307 |
| Ramachandran preferred (%) | 85.31 |
| Ramachandran allowed (%) | 11.41 |
