# Supplementary material for: Structural and functional insights into the first Bacillus thuringiensis vegetative insecticidal protein of the Vpb4 fold, active against western corn rootworm
Source: PLoS One. 2021 Dec 20;16(12):e0260532. doi: 10.1371/journal.pone.0260532 (PMC8687597; doi:10.1371/journal.pone.0260532)
Supplement: S2 Table — a Total number of insects tested per dose. b Means followed by an asterisk are significantly different from untreated control at p_value < 0.04.c Tryptic core (trypsin processed) Vip4Da2. (DOCX) [file pone.0260532.s002.docx]

|  | Test set | Dose (µg/cm^2^) | N^a^ | Mean % mortality (± SD) ^b^ |
| --- | --- | --- | --- | --- |
| Vpb4Da2 | 3 | 11.76 | 32 | 50.00 ± 21.98 * |
|  | 3 | 47.06 | 32 | 87.50 ± 14.43 * |
| Vpb4Da2_Tc^c^ | 3 | 11.76 | 32 | 71.43 ± 20.20 * |
|  | 3 | 47.06 | 32 | 100.00 ± 0.00 * |
| Vpb4Da2_K733C_A422C | 4 | 11.76 | 32 | 59.67 ± 17.74 * |
|  | 4 | 47.06 | 32 | 74.11 ± 20.49 * |
| Vpb4Da2_K733C_A422C-IAF | 4 | 11.76 | 32 | 39.29 ± 19.83* |
|  | 4 | 47.06 | 32 | 70.98 ± 15.59 * |
| Vpb4Da2_T295C_T493C (DIP) | 5 | 11.76 | 24 | 4.76 ± 8.25 |
|  | 5 | 47.06 | 24 | 21.43 ± 25.75 |
| Assay Buffer | 3 | 0.00 | 32 | 7.29 ± 8.59 |
| Assay Buffer | 4 | 0.00 | 32 | 6.70 ± 7.77 |
| Assay Buffer | 5 | 0.00 | 24 | 16.19 ± 3.30 |
