# Supplementary material for: Structural and functional insights into the first Bacillus thuringiensis vegetative insecticidal protein of the Vpb4 fold, active against western corn rootworm
Source: PLoS One. 2021 Dec 20;16(12):e0260532. doi: 10.1371/journal.pone.0260532 (PMC8687597; doi:10.1371/journal.pone.0260532)
Supplement: S3 Table — a Total number of insects used per dose. b Means followed by an asterisk are significantly different from untreated control at p_value < 0.04. (DOCX) [file pone.0260532.s003.docx]

|  |  | Test set | Dose (µg/cm^2^) | N ^a^ | Mean % mortality  (± SD) ^b^ |
| --- | --- | --- | --- | --- | --- |
| Wild-types | Vpb4Da2 | 1 | 14.71 | 32 | 54.46 ± 30.60 * |
|  |  | 1 | 44.12 | 32 | 100.00 ± 0.00 * |
|  | Vpb4C.6693 | 1 | 14.71 | 32 | 4.17 ± 8.34 |
|  |  | 1 | 44.12 | 32 | 3.57 ± 7.15 |
| Chimera-1 | Vpb4Da2_D1-D3 /Vpb4C.6693_D4-D6 | 1 | 14.71 | 32 | 9.82 ± 6.60 |
|  |  | 1 | 44.12 | 32 | 0.00 ± 0.00 |
| Chimera-2 | TIC6693_D1-D3 /Vpb4Da2_D4-D6 | 1 | 14.71 | 32 | 47.62 ± 16.95 * |
|  |  | 1 | 44.12 | 32 | 91.43 ± 10.17 * |
| Chimera-3 | Vpb4Da2_D1-D4, D6/Vpb4C.6693_D5 | 2 | 14.71 | 32 | 17.86 ± 17.98 |
|  |  | 2 | 44.12 | 32 | 23.81 ± 5.05 * |
| Chimera-4 | Vpb4Da2_D1-D5/Vpb4C.6693_D6 | 2 | 14.71 | 32 | 18.75 ± 16.14 |
|  |  | 2 | 41.18 | 32 | 22.32 ± 21.10 |
| Chimera-5 | Vpb4Da2_D1-D4/Vpb4C.6693_D5-D6 | 2 | 14.71 | 32 | 20.54 ± 9.28 |
|  |  | 2 | 44.12 | 32 | 18.75 ± 14.23 |
| Vpb4Da2-Δ1 | Vpb4Da2_D1-D5 | 1 | 14.71 | 32 | 0.00 ± 0.00 |
|  |  | 1 | 44.12 | 32 | 7.14 ± 14.29 |
| Vpb4Da2-Δ2 | Vpb4Da2_D1-D4 | 1 | 14.71 | 32 | 12.50 ± 15.96 |
|  |  | 1 | 44.12 | 32 | 3.57 ± 7.15 |
| Vpb4Da2-Δ3 | Vpb4Da2_D1-D3 | 1 | 14.71 | 32 | 6.25 ± 12.50 |
|  |  | 1 | 44.12 | 32 | 10.72 ± 13.68 |
| Controls | Assay buffer | 1 | 0.00 | 32 | 5.00 ± 10.00 |
|  | Assay buffer | 2 | 0.00 | 32 | 15.03 ± 2.03 |
