# Supplementary figures and images for: Structural and functional insights into the first Bacillus thuringiensis vegetative insecticidal protein of the Vpb4 fold, active against western corn rootworm
Source: PLoS One. 2021 Dec 20;16(12):e0260532. doi: 10.1371/journal.pone.0260532 (PMC8687597; doi:10.1371/journal.pone.0260532)

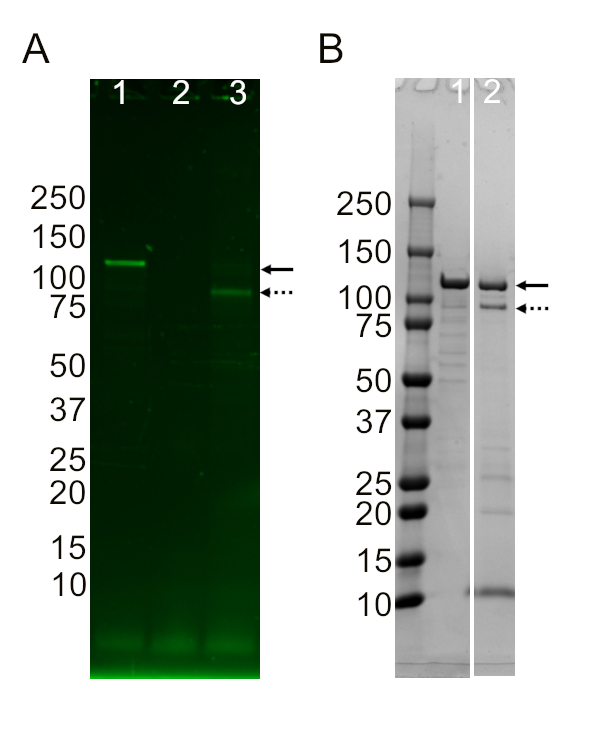

Supplement: S1 Fig — (A) In vivo fate of fluorescently labeled Vpb4Da2 after 24 h feeding of neonate WCR. Lane 1, labeled protein only; lane 2, WCR extract from buffer control treatment; lane 3, WCR extract from larvae treated with labeled Vpb4Da2. (B) In vitro WCR gut-fluid (GF) processing of Vpb4Da2 at pH 6.0. Lane 1 and lane 2 are un-processed full-length (solid arrow) and GF processed Vpb4Da2 (dash arrow), respectively. Protein band at ~10 kDa represents protease inhibitors. (TIF) [file pone.0260532.s004.tif]

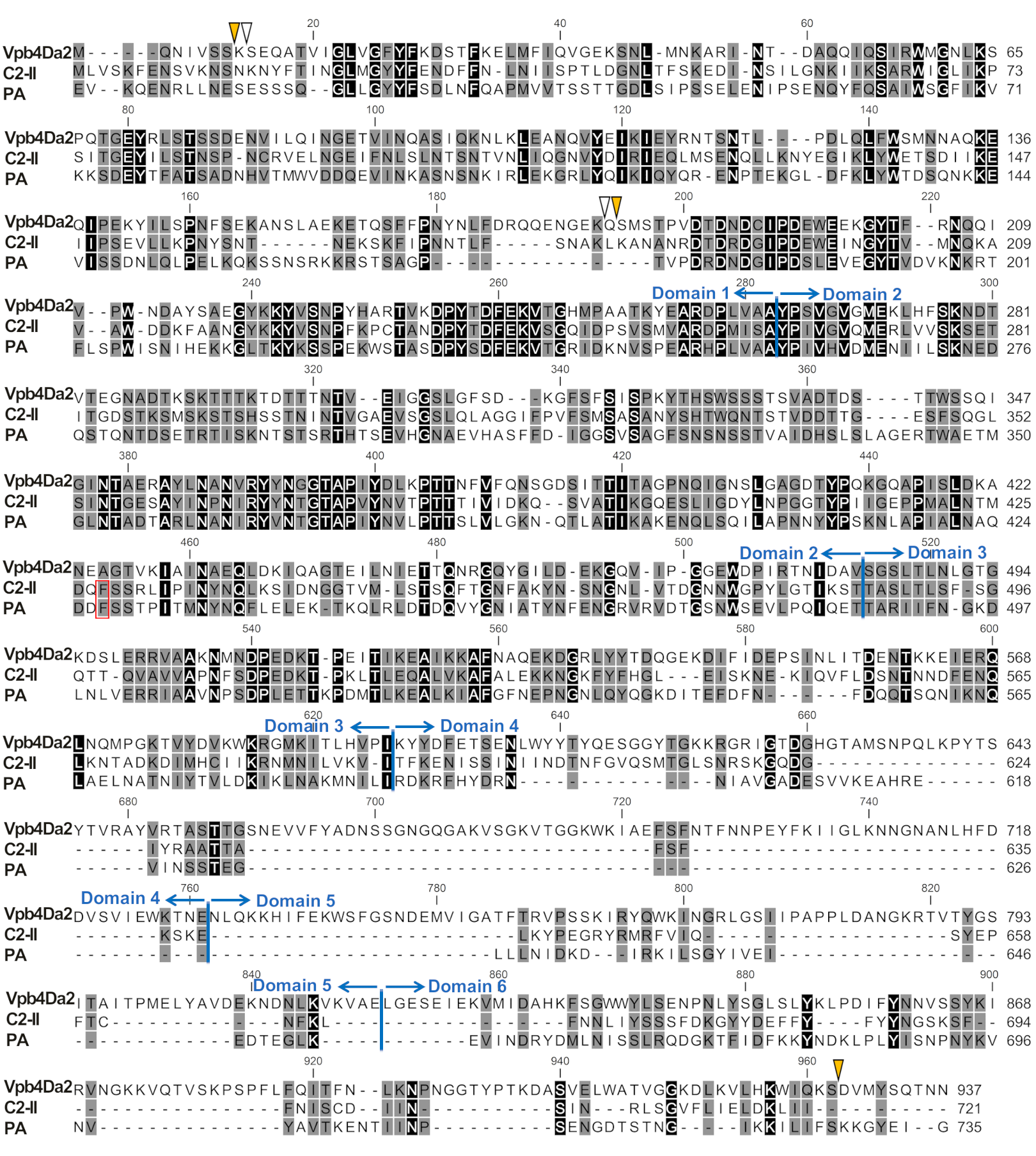

Supplement: S2 Fig — Yellow arrowheads indicate WCR gut fluid cleavage sites while white arrowheads show trypsin cleavage sites. Domain boundaries are delineated in blue. Conserved phenylalanine, Φ-clamps, of C2-II and PA are in the red box. Numbers on sequences are relative to amino acid position 1. Sequence conservation gradient is from conserved (black shade) to diverse (no shade). Sequence alignment was performed using CLCBio™ version 7.6.4. (TIF) [file pone.0260532.s005.tif]

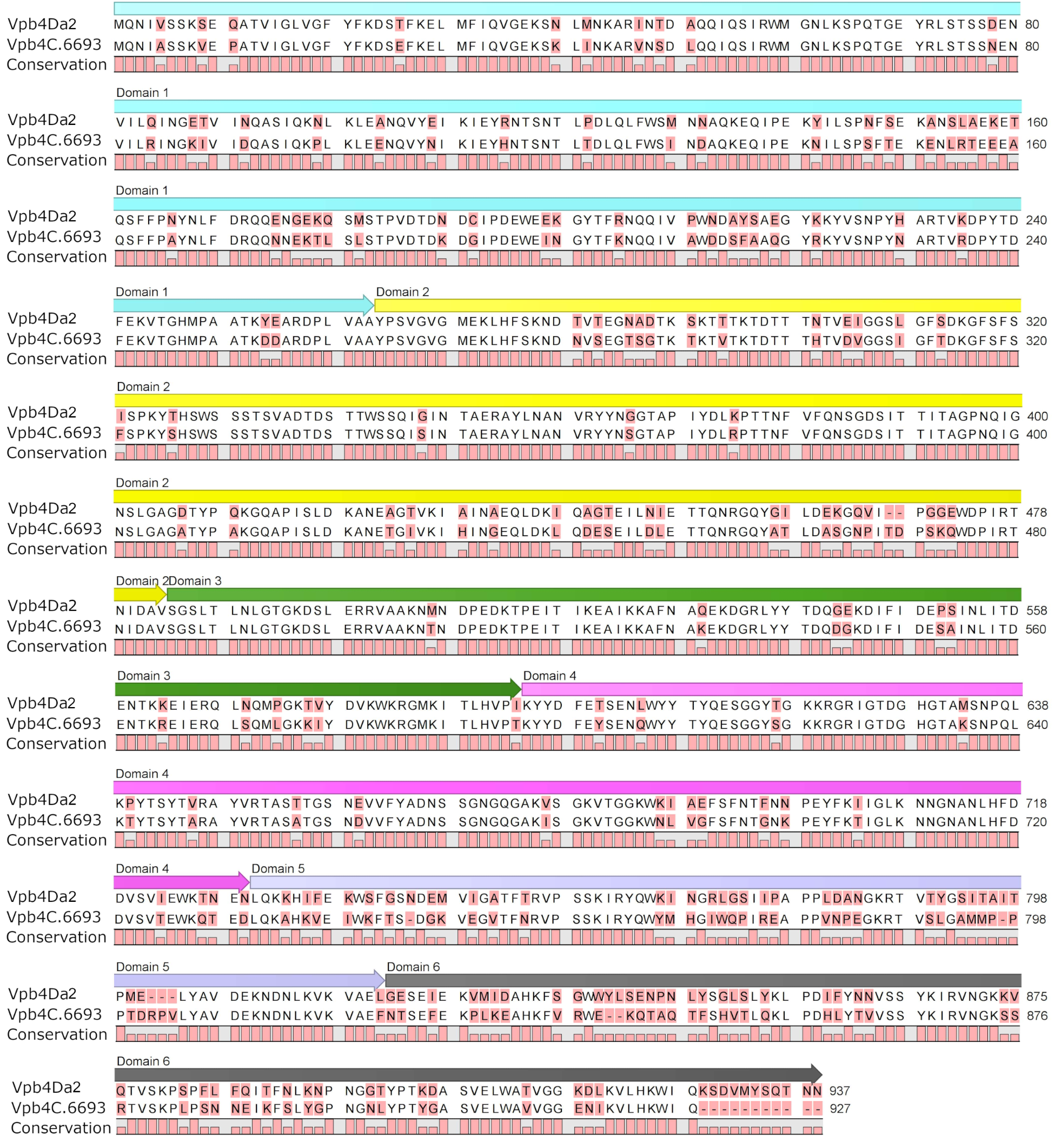

Supplement: S3 Fig — Alignment was obtained using CLCBio™ version 7.6.4. Sequence conservation and domain boundaries are also indicated. (TIF) [file pone.0260532.s006.tif]

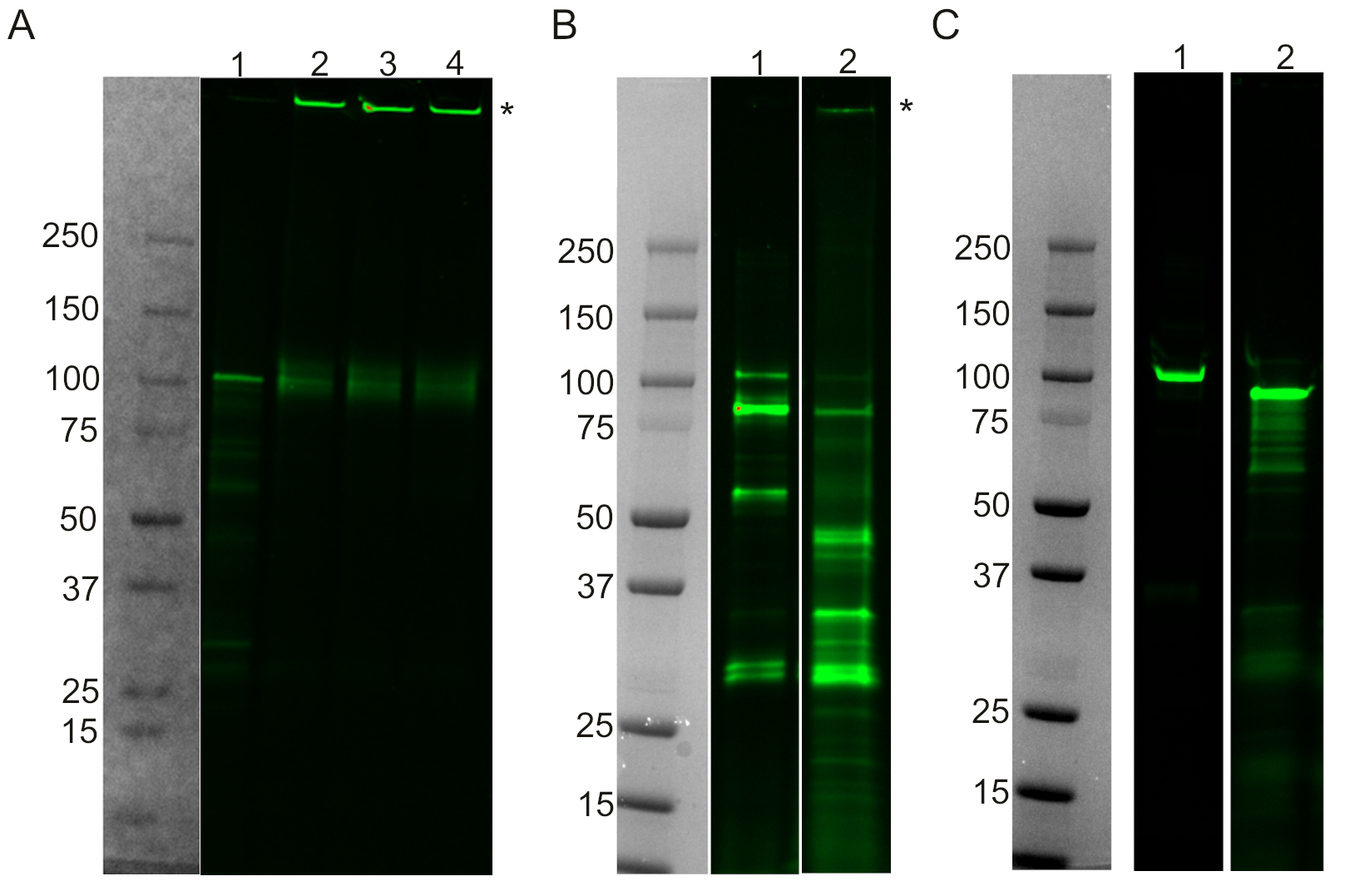

Supplement: S4 Fig — (TIF) [file pone.0260532.s007.tif]

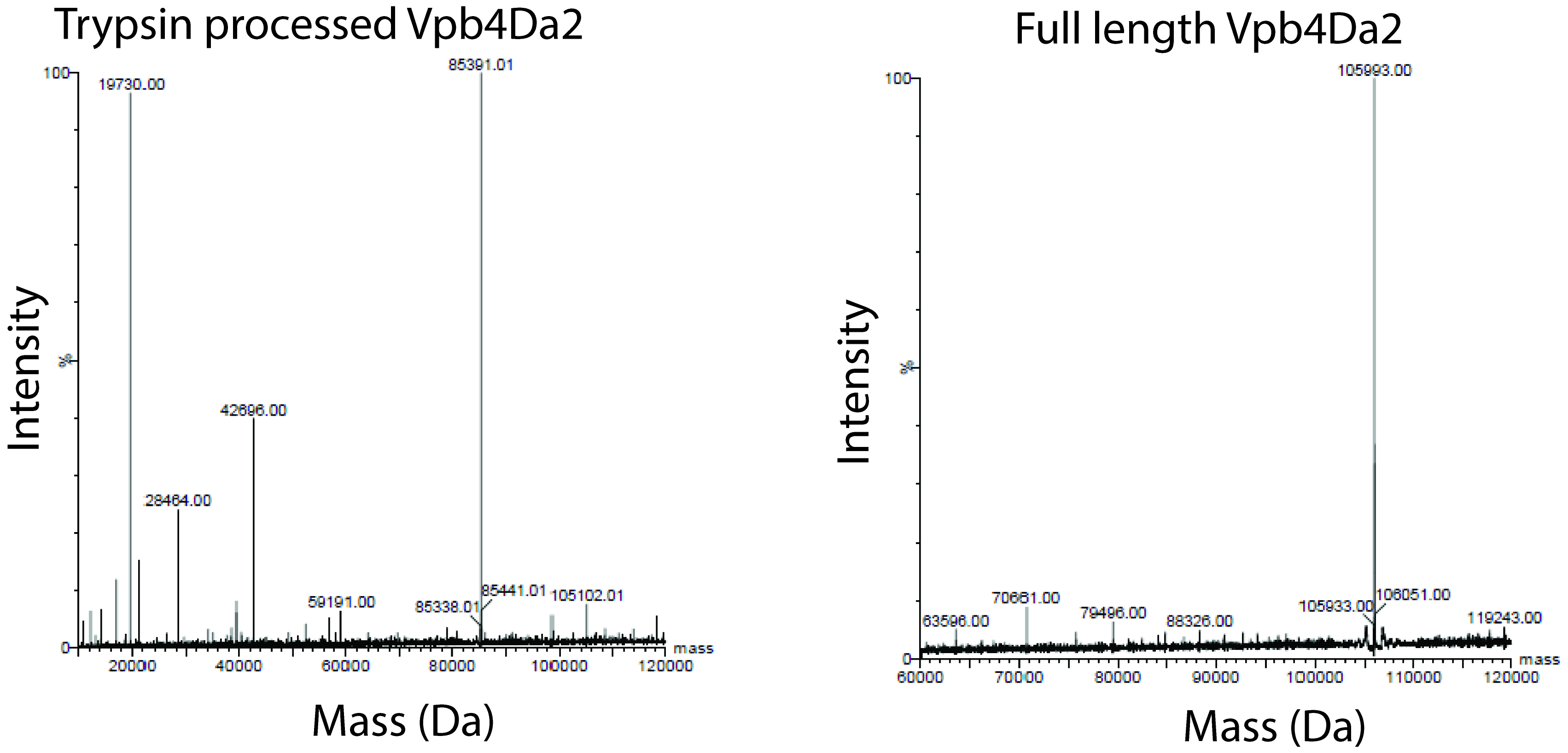

Supplement: S7 File — Molecular masses of proteolytically processed and full length Vpb4Da2 are shown. Fragment of 42,696 Da is the doubly charged form of the 85,391 Da fragment. (TIF) [file pone.0260532.s015.tif]

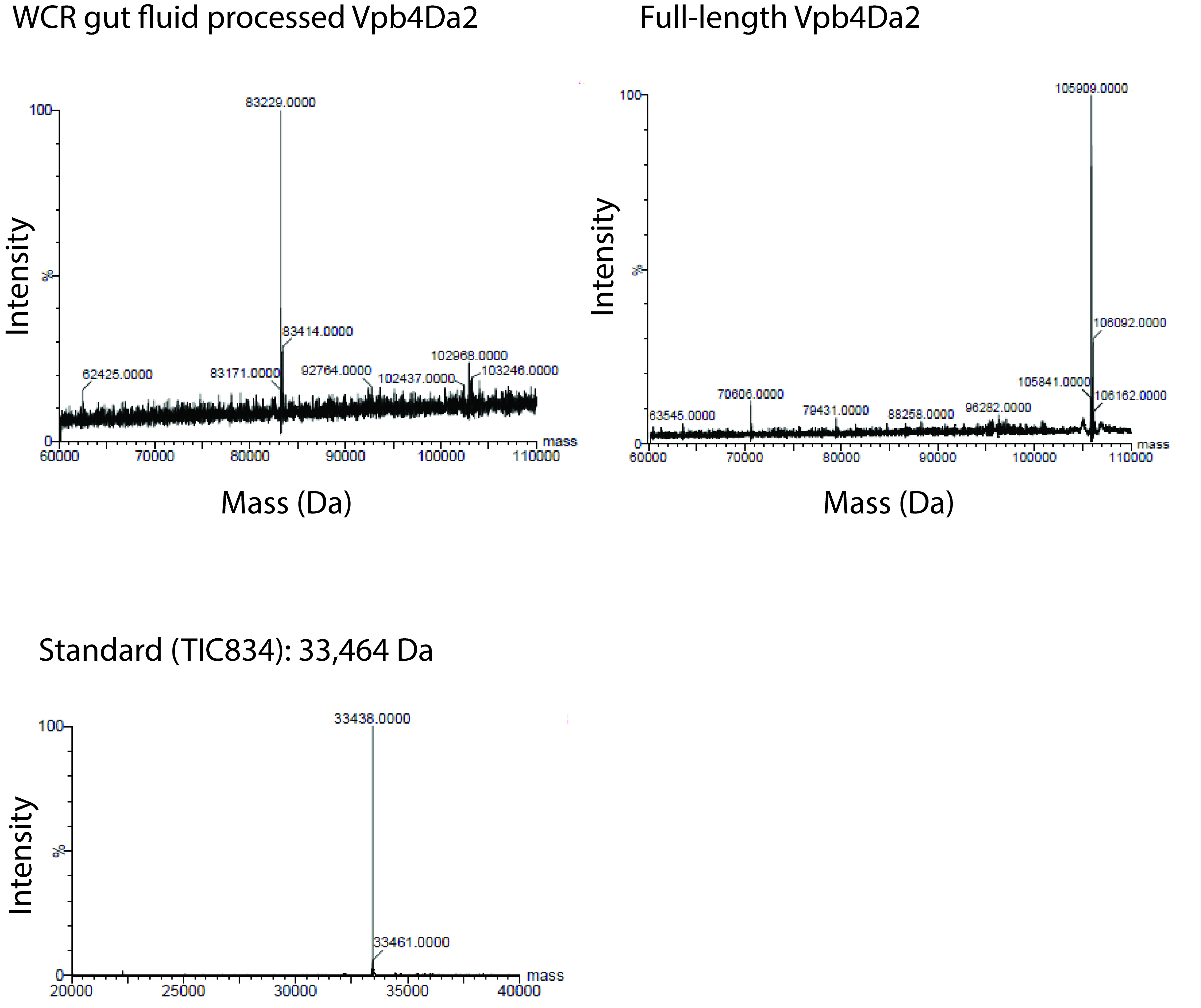

Supplement: S8 File — Molecular masses of proteolytically processed and full length Vpb4Da2 are shown. (TIF) [file pone.0260532.s016.tif]
