# Supplementary material for: Structural and functional insights into the first Bacillus thuringiensis vegetative insecticidal protein of the Vpb4 fold, active against western corn rootworm
Source: PLoS One. 2021 Dec 20;16(12):e0260532. doi: 10.1371/journal.pone.0260532 (PMC8687597; doi:10.1371/journal.pone.0260532)
Supplement: S5 Fig — (PDF) [file pone.0260532.s008.pdf]

Full wwPDB X-ray Structure Validation Report ⓘ

Apr 26, 2021 – 08:02 AM EDT

PDB ID	:	7MJR
Title	:	Vip4Da2 toxin structure
Authors	:	Rydel, T.J.; Duda, D.; Zheng, M.; Henry, A.
Deposited on	:	2021-04-20
Resolution	:	3.22 Å(reported)

This is a Full wwPDB X-ray Structure Validation Report for a publicly released PDB entry.

We welcome your comments at *validation@mail.wwpdb.org*
A user guide is available at
https://www.wwpdb.org/validation/2017/XrayValidationReportHelp
with specific help available everywhere you see the ⓘ symbol.

The following versions of software and data (see references ⓘ) were used in the production of this report:

MolProbity	:	4.02b-467
Mogul	:	1.8.5 (274361), CSD as541be (2020)
Xtriage (Phenix)	:	1.13
EDS	:	2.18
Percentile statistics	:	20191225.v01 (using entries in the PDB archive December 25th 2019)
Refmac	:	5.8.0158
CCP4	:	7.0.044 (Gargrove)
Ideal geometry (proteins)	:	Engh & Huber (2001)
Ideal geometry (DNA, RNA)	:	Parkinson et al. (1996)
Validation Pipeline (wwPDB-VP)	:	2.18

1 Overall quality at a glance (i)

The following experimental techniques were used to determine the structure:
X-RAY DIFFRACTION

The reported resolution of this entry is 3.22 Å.

Percentile scores (ranging between 0-100) for global validation metrics of the entry are shown in the following graphic. The table shows the number of entries on which the scores are based.

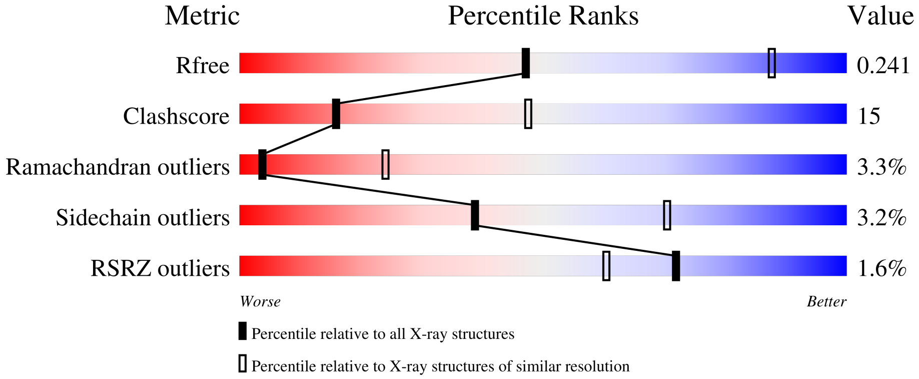

Metric	Whole archive (#Entries)	Similar resolution (#Entries, resolution range(Å))
R_{free}	130704	1335 (3.24-3.20)
Clashscore	141614	1460 (3.24-3.20)
Ramachandran outliers	138981	1437 (3.24-3.20)
Sidechain outliers	138945	1436 (3.24-3.20)
RSRZ outliers	127900	1291 (3.24-3.20)

The table below summarises the geometric issues observed across the polymeric chains and their fit to the electron density. The red, orange, yellow and green segments of the lower bar indicate the fraction of residues that contain outliers for >=3, 2, 1 and 0 types of geometric quality criteria respectively. A grey segment represents the fraction of residues that are not modelled. The numeric value for each fraction is indicated below the corresponding segment, with a dot representing fractions <=5% The upper red bar (where present) indicates the fraction of residues that have poor fit to the electron density. The numeric value is given above the bar.

Mol	Chain	Length	Quality of chain
1	A	937	% 63% 30% • •

2 Entry composition (i)

There are 3 unique types of molecules in this entry. The entry contains 7173 atoms, of which 0 are hydrogens and 0 are deuteriums.

In the tables below, the ZeroOcc column contains the number of atoms modelled with zero occupancy, the AltConf column contains the number of residues with at least one atom in alternate conformation and the Trace column contains the number of residues modelled with at most 2 atoms.

- Molecule 1 is a protein called Vip4Da1 protein.

Mol	Chain	Residues	Atoms					ZeroOcc	AltConf	Trace
			Total	C	N	O	S			
1	A	899	7125	4514	1191	1405	15	0	0	0

There are 3 discrepancies between the modelled and reference sequences:

Chain	Residue	Modelled	Actual	Comment	Reference
A	530	TYR	GLN	engineered mutation	UNP A0A2U5FTM5
A	531	TYR	GLU	engineered mutation	UNP A0A2U5FTM5
A	532	TYR	LYS	engineered mutation	UNP A0A2U5FTM5

- Molecule 2 is CALCIUM ION (three-letter code: CA) (formula: Ca).

Mol	Chain	Residues	Atoms		ZeroOcc	AltConf
			Total	Ca		
2	A	3	3	3	0	0

- Molecule 3 is SULFATE ION (three-letter code: SO4) (formula: O_4S).

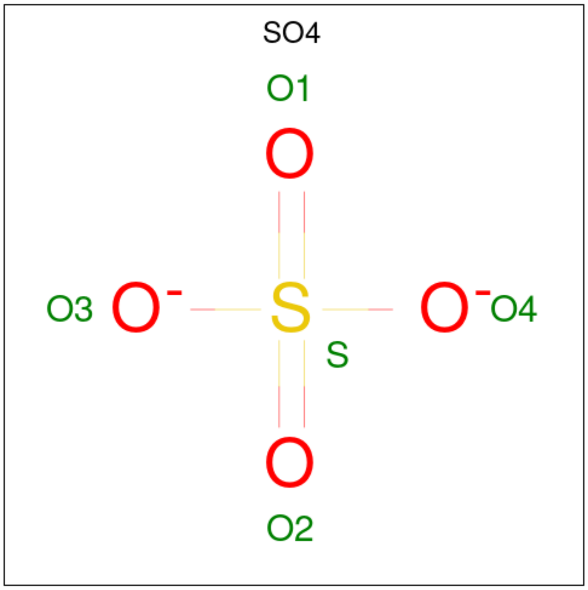

Mol	Chain	Residues	Atoms	ZeroOcc	AltConf
3	A	1	Total O S<br>5 4 1	0	0
3	A	1	Total O S<br>5 4 1	0	0
3	A	1	Total O S<br>5 4 1	0	0
3	A	1	Total O S<br>5 4 1	0	0
3	A	1	Total O S<br>5 4 1	0	0
3	A	1	Total O S<br>5 4 1	0	0
3	A	1	Total O S<br>5 4 1	0	0
3	A	1	Total O S<br>5 4 1	0	0
3	A	1	Total O S<br>5 4 1	0	0

3 Residue-property plots (i)

These plots are drawn for all protein, RNA, DNA and oligosaccharide chains in the entry. The first graphic for a chain summarises the proportions of the various outlier classes displayed in the second graphic. The second graphic shows the sequence view annotated by issues in geometry and electron density. Residues are color-coded according to the number of geometric quality criteria for which they contain at least one outlier: green = 0, yellow = 1, orange = 2 and red = 3 or more. A red dot above a residue indicates a poor fit to the electron density (RSRZ > 2). Stretches of 2 or more consecutive residues without any outlier are shown as a green connector. Residues present in the sample, but not in the model, are shown in grey.

• Molecule 1: Vip4Da1 protein

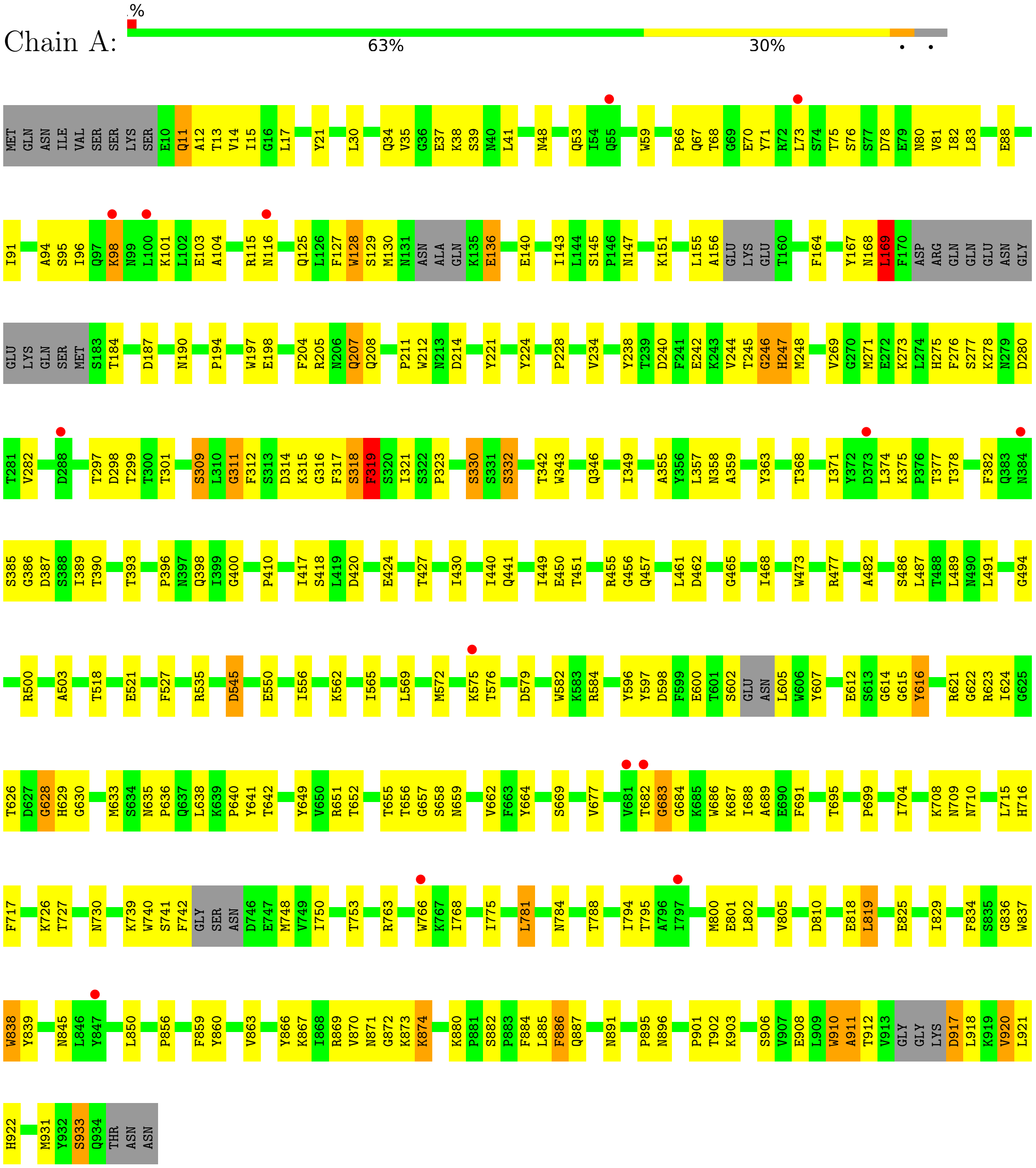

4 Data and refinement statistics (i)

Property	Value	Source
Space group	C 2 2 21	Depositor
Cell constants<br>a, b, c, α, β, γ	117.52Å 221.37Å 154.14Å<br>90.00° 90.00° 90.00°	Depositor
Resolution (Å)	43.05 – 3.22<br>49.19 – 3.22	Depositor<br>EDS
% Data completeness<br>(in resolution range)	93.7 (43.05-3.22)<br>93.1 (49.19-3.22)	Depositor<br>EDS
R_{merge}	0.16	Depositor
R_{sym}	(Not available)	Depositor
$< I/\sigma(I) >$ [1]	3.24 (at 3.25Å)	Xtriage
Refinement program	PHENIX 1.17.1_3660	Depositor
R, R_{free}	0.196 , 0.241<br>0.196 , 0.241	Depositor<br>DCC
R_{free} test set	1926 reflections (6.04%)	wwPDB-VP
Wilson B-factor (Å^2)	91.9	Xtriage
Anisotropy	0.397	Xtriage
Bulk solvent k_{sol}(e/Å^3), B_{sol}(Å^2)	0.30 , 48.3	EDS
L-test for twinning[2]	$< \|L\| > = 0.49$, $< L^2 > = 0.32$	Xtriage
Estimated twinning fraction	No twinning to report.	Xtriage
F_o,F_c correlation	0.93	EDS
Total number of atoms	7173	wwPDB-VP
Average B, all atoms (Å^2)	92.0	wwPDB-VP

Xtriage's analysis on translational NCS is as follows: *The largest off-origin peak in the Patterson function is 3.35% of the height of the origin peak. No significant pseudotranslation is detected.*

[1]Intensities estimated from amplitudes.

[2]Theoretical values of $< |L| >$, $< L^2 >$ for acentric reflections are 0.5, 0.333 respectively for untwinned datasets, and 0.375, 0.2 for perfectly twinned datasets.

5 Model quality (i)

5.1 Standard geometry (i)

Bond lengths and bond angles in the following residue types are not validated in this section: SO4, CA

The Z score for a bond length (or angle) is the number of standard deviations the observed value is removed from the expected value. A bond length (or angle) with $|Z| > 5$ is considered an outlier worth inspection. RMSZ is the root-mean-square of all Z scores of the bond lengths (or angles).

Mol	Chain	Bond lengths		Bond angles	
		RMSZ	#$|Z|$ >5	RMSZ	#$|Z|$ >5
1	A	0.57	1/7284 (0.0%)	0.72	3/9873 (0.0%)

Chiral center outliers are detected by calculating the chiral volume of a chiral center and verifying if the center is modelled as a planar moiety or with the opposite hand.A planarity outlier is detected by checking planarity of atoms in a peptide group, atoms in a mainchain group or atoms of a sidechain that are expected to be planar.

Mol	Chain	#Chirality outliers	#Planarity outliers
1	A	0	3

All (1) bond length outliers are listed below:

Mol	Chain	Res	Type	Atoms	Z	Observed(Å)	Ideal(Å)
1	A	920	VAL	CB-CG1	-8.36	1.35	1.52

All (3) bond angle outliers are listed below:

Mol	Chain	Res	Type	Atoms	Z	Observed(^{o})	Ideal(^{o})
1	A	819	LEU	CA-CB-CG	5.19	127.23	115.30
1	A	921	LEU	CA-CB-CG	5.12	127.08	115.30
1	A	169	LEU	CA-CB-CG	5.07	126.95	115.30

There are no chirality outliers.

All (3) planarity outliers are listed below:

Mol	Chain	Res	Type	Group
1	A	318	SER	Peptide
1	A	884	PHE	Peptide
1	A	901	PRO	Peptide

5.2 Too-close contacts (i)

In the following table, the Non-H and H(model) columns list the number of non-hydrogen atoms and hydrogen atoms in the chain respectively. The H(added) column lists the number of hydrogen atoms added and optimized by MolProbity. The Clashes column lists the number of clashes within the asymmetric unit, whereas Symm-Clashes lists symmetry-related clashes.

Mol	Chain	Non-H	H(model)	H(added)	Clashes	Symm-Clashes
1	A	7125	0	6945	206	0
2	A	3	0	0	0	0
3	A	45	0	0	1	0
All	All	7173	0	6945	206	0

The all-atom clashscore is defined as the number of clashes found per 1000 atoms (including hydrogen atoms). The all-atom clashscore for this structure is 15.

All (206) close contacts within the same asymmetric unit are listed below, sorted by their clash magnitude.

Atom-1	Atom-2	Interatomic distance (Å)	Clash overlap (Å)
1:A:167:TYR:O	1:A:168:ASN:ND2	1.68	1.24
1:A:487:LEU:CD1	1:A:582:TRP:CH2	2.53	0.90
1:A:487:LEU:HD11	1:A:582:TRP:CH2	2.13	0.84
1:A:385:SER:O	1:A:387:ASP:N	2.10	0.83
1:A:801:GLU:HG2	1:A:818:GLU:HG3	1.59	0.83
1:A:870:VAL:HG12	1:A:895:PRO:HB2	1.59	0.82
1:A:688:ILE:HD12	1:A:688:ILE:O	1.80	0.81
1:A:659:ASN:HD22	1:A:709:ASN:HD21	1.30	0.78
1:A:487:LEU:CD1	1:A:582:TRP:HH2	1.96	0.78
1:A:309:SER:HB2	1:A:691:PHE:HA	1.67	0.76
1:A:487:LEU:HD13	1:A:582:TRP:CH2	2.20	0.76
1:A:829:ILE:HD11	1:A:918:LEU:HB3	1.67	0.76
1:A:615:GLY:HA2	1:A:621:ARG:HB3	1.67	0.75
1:A:21:TYR:OH	1:A:37:GLU:OE2	2.05	0.74
1:A:612:GLU:OE2	1:A:621:ARG:NH1	2.21	0.74
1:A:371:ILE:HD11	1:A:456:GLY:HA3	1.68	0.73
1:A:656:THR:O	1:A:658:SER:N	2.22	0.72
1:A:487:LEU:HD11	1:A:582:TRP:CZ3	2.24	0.72
1:A:88:GLU:OE2	1:A:98:LYS:NZ	2.20	0.71
1:A:750:ILE:HG21	1:A:800:MET:HE1	1.72	0.71
1:A:205:ARG:HB2	1:A:221:TYR:CZ	2.27	0.70
1:A:301:THR:HG22	1:A:556:ILE:HD12	1.72	0.69
1:A:277:SER:HB3	1:A:282:VAL:HG22	1.75	0.68
1:A:651:ARG:HG2	1:A:716:HIS:HB2	1.75	0.67

Continued on next page...

Continued from previous page...

Atom-1	Atom-2	Interatomic distance (Å)	Clash overlap (Å)
1:A:866:TYR:CE2	1:A:911:ALA:HB2	2.29	0.67
1:A:67:GLN:O	1:A:71:TYR:OH	2.11	0.67
1:A:688:ILE:HD12	1:A:688:ILE:C	2.14	0.67
1:A:640:PRO:HA	1:A:699:PRO:HG3	1.76	0.66
1:A:343:TRP:CE2	1:A:441:GLN:HB2	2.32	0.65
1:A:377:THR:HG22	1:A:393:THR:HG23	1.77	0.65
1:A:635:ASN:ND2	1:A:704:ILE:HA	2.12	0.65
1:A:659:ASN:ND2	1:A:709:ASN:HD21	1.94	0.64
1:A:197:TRP:CZ2	1:A:211:PRO:HD3	2.33	0.63
1:A:885:LEU:O	1:A:886:PHE:HB3	1.97	0.63
1:A:11:GLN:OE1	1:A:12:ALA:N	2.32	0.63
1:A:298:ASP:OD2	1:A:301:THR:HG23	1.99	0.63
1:A:462:ASP:HB3	1:A:468:ILE:HD12	1.81	0.62
1:A:240:ASP:O	1:A:244:VAL:HG12	1.99	0.62
1:A:918:LEU:O	1:A:920:VAL:HG23	2.00	0.62
1:A:652:THR:HG23	1:A:682:THR:HG22	1.81	0.61
1:A:315:LYS:HG2	1:A:317:PHE:CE1	2.35	0.61
1:A:741:SER:OG	1:A:742:PHE:N	2.33	0.61
1:A:607:TYR:CE2	1:A:633:MET:HG2	2.36	0.61
1:A:78:ASP:HB3	1:A:94:ALA:HB2	1.82	0.60
1:A:635:ASN:HB2	1:A:636:PRO:CD	2.31	0.60
1:A:869:ARG:HD2	1:A:908:GLU:OE2	2.01	0.60
1:A:17:LEU:HD13	1:A:143:ILE:HG23	1.84	0.59
1:A:75:THR:HG21	1:A:81:VAL:HG11	1.84	0.59
1:A:68:THR:HG23	1:A:103:GLU:HA	1.84	0.59
1:A:838:TRP:HH2	1:A:887:GLN:HE22	1.48	0.58
1:A:234:VAL:HG23	1:A:503:ALA:HB2	1.86	0.57
1:A:642:THR:HG23	1:A:727:THR:OG1	2.04	0.57
1:A:655:THR:HG22	1:A:656:THR:HG23	1.85	0.57
1:A:83:LEU:HD23	1:A:91:ILE:HD13	1.87	0.56
1:A:410:PRO:HG3	1:A:417:ILE:HG23	1.86	0.56
1:A:487:LEU:HD13	1:A:582:TRP:HH2	1.63	0.56
1:A:38:LYS:HD2	1:A:140:GLU:HB2	1.86	0.56
1:A:763:ARG:HB2	1:A:805:VAL:HB	1.88	0.55
1:A:212:TRP:CH2	1:A:214:ASP:HA	2.42	0.55
1:A:781:LEU:HD12	1:A:781:LEU:H	1.72	0.55
1:A:398:GLN:NE2	1:A:424:GLU:HA	2.22	0.55
1:A:597:TYR:OH	1:A:635:ASN:HA	2.07	0.54
1:A:382:PHE:HE1	1:A:440:ILE:HD11	1.72	0.54
1:A:246:GLY:O	1:A:248:MET:N	2.36	0.54
1:A:910:TRP:O	1:A:918:LEU:O	2.26	0.54

Continued on next page...

Continued from previous page...

Atom-1	Atom-2	Interatomic distance (Å)	Clash overlap (Å)
1:A:839:TYR:O	1:A:933:SER:HA	2.08	0.54
1:A:709:ASN:ND2	1:A:715:LEU:HB2	2.23	0.53
1:A:649:TYR:O	1:A:717:PHE:HA	2.08	0.53
1:A:151:LYS:O	1:A:190:ASN:HB3	2.08	0.53
1:A:839:TYR:CZ	1:A:933:SER:HB3	2.44	0.52
1:A:874:LYS:H	1:A:874:LYS:HD2	1.74	0.52
1:A:204:PHE:CZ	1:A:207:GLN:HA	2.44	0.52
1:A:282:VAL:HG11	1:A:357:LEU:HA	1.91	0.52
1:A:349:ILE:HD13	1:A:355:ALA:HB2	1.92	0.52
1:A:912:THR:HA	1:A:917:ASP:HA	1.91	0.52
1:A:11:GLN:OE1	1:A:13:THR:HG22	2.10	0.52
1:A:378:THR:HG23	1:A:451:THR:HB	1.92	0.51
1:A:315:LYS:HG3	1:A:575:LYS:HD2	1.93	0.51
1:A:375:LYS:HE3	1:A:424:GLU:OE1	2.10	0.51
1:A:34:GLN:NE2	1:A:35:VAL:O	2.38	0.51
1:A:315:LYS:HG3	1:A:575:LYS:CD	2.41	0.51
1:A:127:PHE:HD1	1:A:136:GLU:O	1.94	0.51
1:A:688:ILE:C	1:A:688:ILE:CD1	2.79	0.51
1:A:866:TYR:CD2	1:A:911:ALA:HB2	2.44	0.51
1:A:753:THR:HG23	1:A:788:THR:HG22	1.94	0.50
1:A:651:ARG:HD3	1:A:686:TRP:CE2	2.47	0.50
1:A:12:ALA:CB	1:A:465:GLY:HA3	2.42	0.50
1:A:187:ASP:HB2	1:A:194:PRO:HA	1.94	0.49
1:A:247:HIS:HA	1:A:461:LEU:HD22	1.95	0.49
1:A:871:ASN:HB2	1:A:896:ASN:OD1	2.13	0.49
1:A:863:VAL:HG11	1:A:866:TYR:CE1	2.47	0.49
1:A:73:LEU:O	1:A:91:ILE:HD11	2.13	0.49
1:A:763:ARG:NH1	1:A:775:ILE:HG21	2.27	0.49
1:A:635:ASN:HB2	1:A:636:PRO:HD3	1.93	0.49
1:A:477:ARG:NH2	3:A:1005:SO4:O4	2.29	0.49
1:A:70:GLU:O	1:A:130:MET:HA	2.13	0.48
1:A:30:LEU:HD12	1:A:164:PHE:HE2	1.77	0.48
1:A:624:ILE:HB	1:A:628:GLY:HA3	1.95	0.48
1:A:271:MET:HE3	1:A:449:ILE:HD11	1.95	0.48
1:A:629:HIS:HE1	1:A:664:TYR:OH	1.96	0.48
1:A:535:ARG:NH2	1:A:598:ASP:OD2	2.47	0.48
1:A:420:ASP:HB2	1:A:427:THR:HG22	1.96	0.48
1:A:572:MET:O	1:A:575:LYS:HG2	2.14	0.48
1:A:839:TYR:CE1	1:A:933:SER:HB3	2.49	0.48
1:A:14:VAL:HG12	1:A:247:HIS:NE2	2.29	0.48
1:A:96:ILE:HG22	1:A:98:LYS:H	1.79	0.48

Continued on next page...

Continued from previous page...

Atom-1	Atom-2	Interatomic distance (Å)	Clash overlap (Å)
1:A:299:THR:OG1	1:A:332:SER:HB2	2.13	0.48
1:A:377:THR:HG22	1:A:393:THR:CG2	2.44	0.48
1:A:607:TYR:CD2	1:A:633:MET:HG2	2.49	0.48
1:A:68:THR:N	1:A:104:ALA:HB2	2.28	0.47
1:A:871:ASN:O	1:A:873:LYS:N	2.47	0.47
1:A:662:VAL:CG2	1:A:708:LYS:HB2	2.44	0.47
1:A:482:ALA:O	1:A:584:ARG:HD3	2.13	0.47
1:A:602:SER:HA	1:A:605:LEU:HD11	1.96	0.47
1:A:342:THR:O	1:A:346:GLN:HG3	2.15	0.47
1:A:641:TYR:CE1	1:A:730:ASN:HA	2.50	0.47
1:A:768:ILE:HD13	1:A:800:MET:HG2	1.96	0.47
1:A:312:PHE:H	1:A:689:ALA:HA	1.80	0.47
1:A:834:PHE:CZ	1:A:836:GLY:HA2	2.50	0.47
1:A:763:ARG:NH1	1:A:810:ASP:OD1	2.39	0.46
1:A:763:ARG:HH11	1:A:775:ILE:HG21	1.80	0.46
1:A:155:LEU:HD13	1:A:156:ALA:N	2.29	0.46
1:A:576:THR:OG1	1:A:579:ASP:OD2	2.29	0.46
1:A:76:SER:OG	1:A:125:GLN:HB2	2.16	0.46
1:A:652:THR:OG1	1:A:684:GLY:N	2.44	0.46
1:A:486:SER:C	1:A:487:LEU:HD12	2.35	0.46
1:A:382:PHE:CZ	1:A:389:ILE:HD11	2.50	0.46
1:A:662:VAL:HG22	1:A:708:LYS:HB2	1.97	0.46
1:A:869:ARG:NH1	1:A:908:GLU:OE1	2.48	0.46
1:A:75:THR:HA	1:A:125:GLN:O	2.15	0.46
1:A:489:LEU:HB2	1:A:527:PHE:CE1	2.50	0.46
1:A:550:GLU:OE1	1:A:576:THR:HB	2.15	0.46
1:A:82:ILE:HG21	1:A:169:LEU:HD12	1.98	0.45
1:A:301:THR:HG22	1:A:556:ILE:CD1	2.43	0.45
1:A:642:THR:CG2	1:A:727:THR:OG1	2.64	0.45
1:A:48:ASN:HB2	1:A:208:GLN:OE1	2.16	0.45
1:A:317:PHE:CE1	1:A:569:LEU:HD13	2.52	0.45
1:A:390:THR:HB	1:A:430:ILE:HG12	1.98	0.45
1:A:115:ARG:HG2	1:A:116:ASN:N	2.32	0.45
1:A:238:TYR:HB3	1:A:242:GLU:HB2	1.98	0.45
1:A:825:GLU:O	1:A:829:ILE:HD13	2.16	0.45
1:A:315:LYS:HG3	1:A:575:LYS:HE3	1.99	0.45
1:A:198:GLU:OE1	1:A:228:PRO:HA	2.16	0.45
1:A:596:TYR:CZ	1:A:598:ASP:HB3	2.52	0.45
1:A:314:ASP:OD2	1:A:687:LYS:HG2	2.17	0.45
1:A:128:TRP:HB2	1:A:136:GLU:HB3	1.99	0.45
1:A:794:ILE:HD12	1:A:794:ILE:HA	1.81	0.44

Continued on next page...

Continued from previous page...

Atom-1	Atom-2	Interatomic distance (Å)	Clash overlap (Å)
1:A:205:ARG:HB2	1:A:221:TYR:CE2	2.51	0.44
1:A:318:SER:OG	1:A:319:PHE:N	2.50	0.44
1:A:297:THR:HG23	1:A:332:SER:HB3	1.99	0.44
1:A:837:TRP:CZ3	1:A:850:LEU:HD23	2.52	0.44
1:A:491:LEU:HD12	1:A:491:LEU:O	2.18	0.44
1:A:629:HIS:NE2	1:A:669:SER:HB3	2.33	0.44
1:A:96:ILE:CG2	1:A:98:LYS:HB2	2.48	0.44
1:A:518:THR:HG23	1:A:521:GLU:OE2	2.18	0.44
1:A:856:PRO:HG2	1:A:859:PHE:CD2	2.53	0.44
1:A:15:ILE:HA	1:A:145:SER:OG	2.17	0.44
1:A:273:LYS:HB2	1:A:273:LYS:HE3	1.79	0.44
1:A:845:ASN:OD1	1:A:903:LYS:HE3	2.18	0.43
1:A:80:ASN:HB3	1:A:115:ARG:O	2.18	0.43
1:A:766:TRP:CE2	1:A:802:LEU:HD13	2.54	0.43
1:A:860:TYR:HE1	1:A:880:LYS:HG2	1.84	0.43
1:A:311:GLY:HA3	1:A:677:VAL:HG11	2.00	0.43
1:A:246:GLY:C	1:A:248:MET:H	2.21	0.43
1:A:319:PHE:HE2	1:A:565:ILE:HG21	1.83	0.43
1:A:269:VAL:HB	1:A:363:TYR:CD1	2.54	0.43
1:A:626:THR:O	1:A:710:ASN:HA	2.19	0.43
1:A:276:PHE:CZ	1:A:343:TRP:HB3	2.54	0.43
1:A:278:LYS:HG3	1:A:349:ILE:HG22	2.00	0.43
1:A:330:SER:OG	1:A:450:GLU:HA	2.18	0.43
1:A:739:LYS:HG2	1:A:753:THR:HB	2.00	0.43
1:A:636:PRO:HG2	1:A:638:LEU:HD11	2.01	0.43
1:A:96:ILE:HG21	1:A:98:LYS:HB2	2.01	0.42
1:A:870:VAL:HA	1:A:906:SER:O	2.19	0.42
1:A:197:TRP:CH2	1:A:211:PRO:HD3	2.54	0.42
1:A:462:ASP:CB	1:A:468:ILE:HD12	2.48	0.42
1:A:659:ASN:OD1	1:A:659:ASN:N	2.52	0.42
1:A:224:TYR:CE2	1:A:244:VAL:HG23	2.54	0.42
1:A:368:THR:HG22	1:A:500:ARG:CZ	2.49	0.42
1:A:652:THR:HG21	1:A:683:GLY:HA2	2.01	0.42
1:A:664:TYR:CZ	1:A:669:SER:HA	2.55	0.42
1:A:750:ILE:HG21	1:A:800:MET:CE	2.47	0.42
1:A:358:ASN:OD1	1:A:359:ALA:N	2.50	0.42
1:A:740:TRP:CZ2	1:A:819:LEU:HB2	2.54	0.42
1:A:385:SER:O	1:A:385:SER:OG	2.28	0.42
1:A:545:ASP:OD1	1:A:545:ASP:N	2.53	0.42
1:A:748:MET:HE2	1:A:795:THR:HA	2.01	0.42
1:A:607:TYR:O	1:A:630:GLY:HA2	2.19	0.42

Continued on next page...

Continued from previous page...

Atom-1	Atom-2	Interatomic distance (Å)	Clash overlap (Å)
1:A:829:ILE:HD11	1:A:918:LEU:HD22	2.01	0.42
1:A:615:GLY:CA	1:A:621:ARG:HB3	2.44	0.41
1:A:867:LYS:HB2	1:A:910:TRP:HB2	2.02	0.41
1:A:53:GLN:HG3	1:A:53:GLN:O	2.21	0.41
1:A:321:ILE:HD12	1:A:562:LYS:HB2	2.01	0.41
1:A:614:GLY:HA3	1:A:621:ARG:NE	2.35	0.41
1:A:371:ILE:HG13	1:A:473:TRP:CD1	2.56	0.41
1:A:41:LEU:HD13	1:A:59:TRP:CD2	2.55	0.41
1:A:396:PRO:HA	1:A:400:GLY:O	2.20	0.41
1:A:624:ILE:HD12	1:A:628:GLY:O	2.20	0.41
1:A:616:TYR:CD1	1:A:616:TYR:C	2.94	0.41
1:A:695:THR:HB	1:A:699:PRO:HA	2.03	0.41
1:A:12:ALA:HB1	1:A:15:ILE:HD13	2.03	0.40
1:A:101:LYS:HE3	1:A:101:LYS:HB3	1.88	0.40
1:A:371:ILE:HG13	1:A:473:TRP:HD1	1.86	0.40
1:A:886:PHE:CD1	1:A:887:GLN:HG2	2.57	0.40
1:A:418:SER:HB2	1:A:420:ASP:OD1	2.22	0.40
1:A:616:TYR:C	1:A:616:TYR:HD1	2.25	0.40

There are no symmetry-related clashes.

5.3 Torsion angles (i)

5.3.1 Protein backbone (i)

In the following table, the Percentiles column shows the percent Ramachandran outliers of the chain as a percentile score with respect to all X-ray entries followed by that with respect to entries of similar resolution.

The Analysed column shows the number of residues for which the backbone conformation was analysed, and the total number of residues.

Mol	Chain	Analysed	Favoured	Allowed	Outliers	Percentiles	
1	A	885/937 (94%)	755 (85%)	101 (11%)	29 (3%)	4	24

All (29) Ramachandran outliers are listed below:

Mol	Chain	Res	Type
1	A	39	SER
1	A	98	LYS
1	A	316	GLY

Continued on next page...

Continued from previous page...

Mol	Chain	Res	Type
1	A	386	GLY
1	A	657	GLY
1	A	902	THR
1	A	129	SER
1	A	245	THR
1	A	311	GLY
1	A	600	GLU
1	A	622	GLY
1	A	628	GLY
1	A	872	GLY
1	A	886	PHE
1	A	247	HIS
1	A	309	SER
1	A	319	PHE
1	A	911	ALA
1	A	184	THR
1	A	207	GLN
1	A	494	GLY
1	A	784	ASN
1	A	136	GLU
1	A	280	ASP
1	A	726	LYS
1	A	246	GLY
1	A	66	PRO
1	A	323	PRO
1	A	683	GLY

5.3.2 Protein sidechains (i)

In the following table, the Percentiles column shows the percent sidechain outliers of the chain as a percentile score with respect to all X-ray entries followed by that with respect to entries of similar resolution.

The Analysed column shows the number of residues for which the sidechain conformation was analysed, and the total number of residues.

Mol	Chain	Analysed	Rotameric	Outliers	Percentiles
1	A	782/815 (96%)	757 (97%)	25 (3%)	39 70

All (25) residues with a non-rotameric sidechain are listed below:

Mol	Chain	Res	Type
1	A	11	GLN
1	A	95	SER
1	A	128	TRP
1	A	147	ASN
1	A	169	LEU
1	A	275	HIS
1	A	319	PHE
1	A	330	SER
1	A	332	SER
1	A	374	LEU
1	A	455	ARG
1	A	457	GLN
1	A	545	ASP
1	A	616	TYR
1	A	623	ARG
1	A	781	LEU
1	A	838	TRP
1	A	874	LYS
1	A	882	SER
1	A	891	ASN
1	A	910	TRP
1	A	917	ASP
1	A	922	HIS
1	A	931	MET
1	A	933	SER

Sometimes sidechains can be flipped to improve hydrogen bonding and reduce clashes. All (2) such sidechains are listed below:

Mol	Chain	Res	Type
1	A	247	HIS
1	A	709	ASN

5.3.3 RNA (i)

There are no RNA molecules in this entry.

5.4 Non-standard residues in protein, DNA, RNA chains (i)

There are no non-standard protein/DNA/RNA residues in this entry.

5.5 Carbohydrates (i)

There are no monosaccharides in this entry.

5.6 Ligand geometry (i)

Of 12 ligands modelled in this entry, 3 are monoatomic - leaving 9 for Mogul analysis.

In the following table, the Counts columns list the number of bonds (or angles) for which Mogul statistics could be retrieved, the number of bonds (or angles) that are observed in the model and the number of bonds (or angles) that are defined in the Chemical Component Dictionary. The Link column lists molecule types, if any, to which the group is linked. The Z score for a bond length (or angle) is the number of standard deviations the observed value is removed from the expected value. A bond length (or angle) with $|Z| > 2$ is considered an outlier worth inspection. RMSZ is the root-mean-square of all Z scores of the bond lengths (or angles).

Mol	Type	Chain	Res	Link	Bond lengths			Bond angles		
					Counts	RMSZ	$\#\|Z\| > 2$	Counts	RMSZ	$\#\|Z\| > 2$
3	SO4	A	1004	-	4,4,4	0.20	0	6,6,6	0.17	0
3	SO4	A	1005	-	4,4,4	0.23	0	6,6,6	0.28	0
3	SO4	A	1007	-	4,4,4	0.19	0	6,6,6	0.39	0
3	SO4	A	1010	-	4,4,4	0.22	0	6,6,6	0.23	0
3	SO4	A	1008	-	4,4,4	0.13	0	6,6,6	0.37	0
3	SO4	A	1011	-	4,4,4	0.22	0	6,6,6	0.11	0
3	SO4	A	1006	-	4,4,4	0.27	0	6,6,6	0.18	0
3	SO4	A	1012	-	4,4,4	0.17	0	6,6,6	0.22	0
3	SO4	A	1009	-	4,4,4	0.20	0	6,6,6	0.18	0

There are no bond length outliers.

There are no bond angle outliers.

There are no chirality outliers.

There are no torsion outliers.

There are no ring outliers.

1 monomer is involved in 1 short contact:

Mol	Chain	Res	Type	Clashes	Symm-Clashes
3	A	1005	SO4	1	0

5.7 Other polymers (i)

There are no such residues in this entry.

5.8 Polymer linkage issues (i)

There are no chain breaks in this entry.

6 Fit of model and data (i)

6.1 Protein, DNA and RNA chains (i)

In the following table, the column labelled '#RSRZ> 2' contains the number (and percentage) of RSRZ outliers, followed by percent RSRZ outliers for the chain as percentile scores relative to all X-ray entries and entries of similar resolution. The OWAB column contains the minimum, median, 95th percentile and maximum values of the occupancy-weighted average B-factor per residue. The column labelled 'Q< 0.9' lists the number of (and percentage) of residues with an average occupancy less than 0.9.

Mol	Chain	Analysed	<RSRZ>	#RSRZ>2			OWAB(Å^2)	Q<0.9
1	A	899/937 (95%)	0.18	14 (1%)	72	60	61, 89, 124, 147	0

All (14) RSRZ outliers are listed below:

Mol	Chain	Res	Type	RSRZ
1	A	682	THR	4.2
1	A	73	LEU	3.2
1	A	288	ASP	3.1
1	A	98	LYS	3.1
1	A	681	VAL	2.9
1	A	384	ASN	2.5
1	A	766	TRP	2.5
1	A	116	ASN	2.5
1	A	847	TYR	2.4
1	A	797	ILE	2.4
1	A	575	LYS	2.1
1	A	100	LEU	2.1
1	A	373	ASP	2.1
1	A	55	GLN	2.1

6.2 Non-standard residues in protein, DNA, RNA chains (i)

There are no non-standard protein/DNA/RNA residues in this entry.

6.3 Carbohydrates (i)

There are no monosaccharides in this entry.

6.4 Ligands (i)

In the following table, the Atoms column lists the number of modelled atoms in the group and the number defined in the chemical component dictionary. The B-factors column lists the minimum, median, 95th percentile and maximum values of B factors of atoms in the group. The column labelled 'Q< 0.9' lists the number of atoms with occupancy less than 0.9.

Mol	Type	Chain	Res	Atoms	RSCC	RSR	B-factors($Å^2$)	Q<0.9
3	SO4	A	1005	5/5	0.83	0.31	113,124,133,137	0
3	SO4	A	1012	5/5	0.85	0.20	131,133,153,153	0
3	SO4	A	1007	5/5	0.89	0.16	109,112,124,127	0
3	SO4	A	1008	5/5	0.90	0.16	99,107,122,124	0
3	SO4	A	1011	5/5	0.90	0.38	133,142,151,158	0
3	SO4	A	1006	5/5	0.90	0.22	118,124,132,148	0
3	SO4	A	1010	5/5	0.92	0.50	125,128,131,142	0
3	SO4	A	1009	5/5	0.94	0.21	117,130,136,146	0
3	SO4	A	1004	5/5	0.95	0.16	103,109,116,120	0
2	CA	A	1003	1/1	0.98	0.16	95,95,95,95	0
2	CA	A	1001	1/1	0.99	0.22	90,90,90,90	0
2	CA	A	1002	1/1	1.00	0.25	81,81,81,81	0

6.5 Other polymers (i)

There are no such residues in this entry.
