# Supplementary material for: Structural and functional insights into the first Bacillus thuringiensis vegetative insecticidal protein of the Vpb4 fold, active against western corn rootworm
Source: PLoS One. 2021 Dec 20;16(12):e0260532. doi: 10.1371/journal.pone.0260532 (PMC8687597; doi:10.1371/journal.pone.0260532)
Supplement: S4 File — (DOCX) [file pone.0260532.s012.docx]

|  | **Mean** | **STD** | **SE** |  |
| --- | --- | --- | --- | --- |
| Vpb4Da2 | 50.00 | 0.00 | 0.00 |  |
| Vpb4C.6693 | 54.00 | 0.33 | 0.19 |  |
| Chimera 1 | 54.75 | 0.12 | 0.08 |  |
| Chimera 2 | 48.58 | 4.33 | 2.17 |  |
| Chimera 3 | 52.50 | 0.47 | 0.33 |  |
| Chimera 4 | 49.58 | 0.12 | 0.08 |  |
| Chimera 5 | 54.17 | 0.71 | 0.50 |  |
|  |  |  |  |  |
|  | Set 1 | Set 2 | Set 3 |  |
| Vpb4Da2 | 50 | 50 | 50 |  |
| Vpb4Da2 | 50 | 50 | 50 |  |
| Vpb4Da2 | 50 | 50 | 50 |  |
|  | Set 1 | Set 2 | Set 3 |  |
| Vpb4C. 6693 | 54 | 55 | 54 |  |
| Vpb4C. 6693 | 54 | 55 | 53 |  |
| Vpb4C. 6693 | 54 | 53 | 54 |  |
|  | Set 1 | Set 2 |  |  |
| Chimera 1 | 54.5 | 54.5 |  |  |
| Chimera 1 | 54.5 | 55 |  |  |
| Chimera 1 | 55 | 55 |  |  |
|  | Set 1 | Set 2 | Set 3 | set 4 |
| Chimera 2 | 51.5 | 49 | 52 | 40 |
| Chimera 2 | 51.5 | 49 | 52 | 47 |
| Chimera 2 | 51 | 49 | 51 | 40 |
|  | Set 1 | Set 2 |  |  |
| Chimera 3 | 52 | 53.5 |  |  |
| Chimera 3 | 52 | 52 |  |  |
| Chimera 3 | 52.5 | 53 |  |  |
|  | Set 1 | Set 2 |  |  |
| Chimera 4 | 49.5 | 49.5 |  |  |
| Chimera 4 | 49.5 | 49.5 |  |  |
| Chimera 4 | 49.5 | 50 |  |  |
|  | Set 1 | Set 2 |  |  |
| Chimera 5 | 53.5 | 54.5 |  |  |
| Chimera 5 | 54 | 54.5 |  |  |
| Chimera 5 | 53.5 | 55 |  |  |
