# Supplementary material for: Structural and functional insights into the first Bacillus thuringiensis vegetative insecticidal protein of the Vpb4 fold, active against western corn rootworm
Source: PLoS One. 2021 Dec 20;16(12):e0260532. doi: 10.1371/journal.pone.0260532 (PMC8687597; doi:10.1371/journal.pone.0260532)
Supplement: S5 File — a Means followed by asterisks are significantly different from buffer control treatment at p-value < 0.040. Dunnett’s test was used. (DOCX) [file pone.0260532.s013.docx]

| **Sample** | **Vpb4Da2 (µg/cm^2^)** | **DIP (µg/cm^2^)** | **N** | **Mean^a^** | **Std Dev** |
| --- | --- | --- | --- | --- | --- |
| Vpb4Da2 only | 23.5 | 0 | 32 | 100.00* | 0.00 |
| 1:1 ratio | 23.5 | 23.5 | 32 | 57.59* | 16.78 |
| 1:10 ratio | 23.5 | 235.3 | 32 | 11.25 | 13.15 |
| DIP variant | 0 | 270.6 | 32 | 10.27 | 13.55 |
| Buffer control | 0 | 0 | 32 | 10.42 | 12.50 |
