# Supplementary material for: Structural and functional insights into the first Bacillus thuringiensis vegetative insecticidal protein of the Vpb4 fold, active against western corn rootworm
Source: PLoS One. 2021 Dec 20;16(12):e0260532. doi: 10.1371/journal.pone.0260532 (PMC8687597; doi:10.1371/journal.pone.0260532)
Supplement: S6 File — a Means followed by asterisks are significantly different from buffer control treatment at p-value < 0.040. Dunnett’s test was used. (DOCX) [file pone.0260532.s014.docx]

| **Sample Name** | **Concentration (µg/Cm^2^)** | **N** | **Mean^a^** | **Std Dev** |
| --- | --- | --- | --- | --- |
| Vpb4Da2 | 1.8 | 24 | 0.00 | 0.00 |
| Vpb4Da2 | 3.7 | 24 | 13.10 | 12.54 |
| Vpb4Da2 | 7.4 | 24 | 11.11 | 9.62 |
| Vpb4Da2 | 14.7 | 24 | 63.89* | 12.73 |
| Vpb4Da2 | 29.4 | 24 | 94.44* | 9.62 |
| Vpb4Da2 | 58.8 | 24 | 100.00* | 0.00 |
| Vpb4Da2 | 117.6 | 24 | 100.00* | 0.00 |
| Buffer control | 0.00 | 24 | 5.56 | 9.62 |
