# Supplementary material for: Structural and functional insights into the first Bacillus thuringiensis vegetative insecticidal protein of the Vpb4 fold, active against western corn rootworm
Source: PLoS One. 2021 Dec 20;16(12):e0260532. doi: 10.1371/journal.pone.0260532 (PMC8687597; doi:10.1371/journal.pone.0260532)
Supplement: S9 File — (DOCX) [file pone.0260532.s017.docx]

>Vpb4Da2_pMON346342

MQNIVSSKSEQATVIGLVGFYFKDSTFKELMFIQVGEKSNLMNKARINTDAQQIQSIRWMGNLKSPQTGEYRLSTSSDENVILQINGETVINQASIQKNLKLEANQVYEIKIEYRNTSNTLPDLQLFWSMNNAQKEQIPEKYILSPNFSEKANSLAEKETQSFFPNYNLFDRQQENGEKQSMSTPVDTDNDCIPDEWEEKGYTFRNQQIVPWNDAYSAEGYKKYVSNPYHARTVKDPYTDFEKVTGHMPAATKYEARDPLVAAYPSVGVGMEKLHFSKNDTVTEGNADTKSKTTTKTDTTTNTVEIGGSLGFSDKGFSFSISPKYTHSWSSSTSVADTDSTTWSSQIGINTAERAYLNANVRYYNGGTAPIYDLKPTTNFVFQNSGDSITTITAGPNQIGNSLGAGDTYPQKGQAPISLDKANEAGTVKIAINAEQLDKIQAGTEILNIETTQNRGQYGILDEKGQVIPGGEWDPIRTNIDAVSGSLTLNLGTGKDSLERRVAAKNMNDPEDKTPEITIKEAIKKAFNAQEKDGRLYYTDQGEKDIFIDEPSINLITDENTKKEIERQLNQMPGKTVYDVKWKRGMKITLHVPIKYYDFETSENLWYYTYQESGGYTGKKRGRIGTDGHGTAMSNPQLKPYTSYTVRAYVRTASTTGSNEVVFYADNSSGNGQGAKVSGKVTGGKWKIAEFSFNTFNNPEYFKIIGLKNNGNANLHFDDVSVIEWKTNENLQKKHIFEKWSFGSNDEMVIGATFTRVPSSKIRYQWKINGRLGSIIPAPPLDANGKRTVTYGSITAITPMELYAVDEKNDNLKVKVAELGESEIEKVMIDAHKFSGWWYLSENPNLYSGLSLYKLPDIFYNNVSSYKIRVNGKKVQTVSKPSPFLFQITFNLKNPNGGTYPTKDASVELWATVGGKDLKVLHKWIQKSDVMYSQTNNHHHHAHHH*

>Vpb4C.6693_pMON342343

MQNIASSKVEPATVIGLVGFYFKDSEFKELMFIQVGEKSKLINKARVNSDLQQIQSIRWMGNLKSPQTGEYRLSTSSNENVILRINGKIVIDQASIQKPLKLEENQVYNIKIEYHNTSNTLTDLQLFWSINDAQKEQIPEKNILSPSFTEKENLRTEEEAQSFFPAYNLFDRQQNNEKTLSLSTPVDTDKDGIPDEWEINGYTFKNQQIVAWDDSFAAQGYRKYVSNPYNARTVRDPYTDFEKVTGHMPAATKDDARDPLVAAYPSVGVGMEKLHFSKNDNVSEGTSGTKTKTVTKTDTTTHTVDVGGSIGFTDKGFSFSFSPKYSHSWSSSTSVADTDSTTWSSQISINTAERAYLNANVRYYNSGTAPIYDLRPTTNFVFQNSGDSITTITAGPNQIGNSLGAGATYPAKGQAPISLDKANETGIVKIHINGEQLDKLQDESEILDLETTQNRGQYATLDASGNPITDPSKQWDPIRTNIDAVSGSLTLNLGTGKDSLERRVAAKNTNDPEDKTPEITIKEAIKKAFNAKEKDGRLYYTDQDGKDIFIDESAINLITDENTKREIERQLSQMLGKKIYDVKWKRGMKITLHVPTKYYDFEYSENQWYYTYQESGGYSGKKRGRIGTDGHGTAKSNPQLKTYTSYTARAYVRTASATGSNDVVFYADNSSGNGQGAKISGKVTGGKWNLVGFSFNTGNKPEYFKTIGLKNNGNANLHFDDVSVTEWKQTEDLQKAHKVEIWKFTSDGKVEGVTFNRVPSSKIRYQWYMHGIWQPIREAPPVNPEGKRTVSLGAMMPPPTDRPVLYAVDEKNDNLKVKVAEFNTSEFEKPLKEAHKFVRWEKQTAQTFSHVTLQKLPDHLYTVVSSYKIRVNGKSSRTVSKPLPSNNEIKFSLYGPNGNLYPTYGASVELWAVVGGENIKVLHKWIQHHHHAHHH*
